# Supplementary material for: Trainable bioinspired magnetic sensitivity adaptation using ferromagnetic colloidal assemblies
Source: Cell Rep Phys Sci. 2024 Apr 17;5(4):101923. doi: 10.1016/j.xcrp.2024.101923 (PMC11043831; doi:10.1016/j.xcrp.2024.101923)
Supplement: Document S1. Figures S1–S17 and Table S1 [file mmc1.pdf]

Cell Reports Physical Science, Volume *5*

Supplemental information

Trainable bioinspired magnetic sensitivity adaptation using ferromagnetic colloidal assemblies

Xianhu Liu, Hongwei Tan, Emil Stråka, Xichen Hu, Min Chen, Sebastiaan van Dijken, Alberto Scacchi, Maria Sammalkorpi, Olli Ikkala, and Bo Peng

Supplemental Information

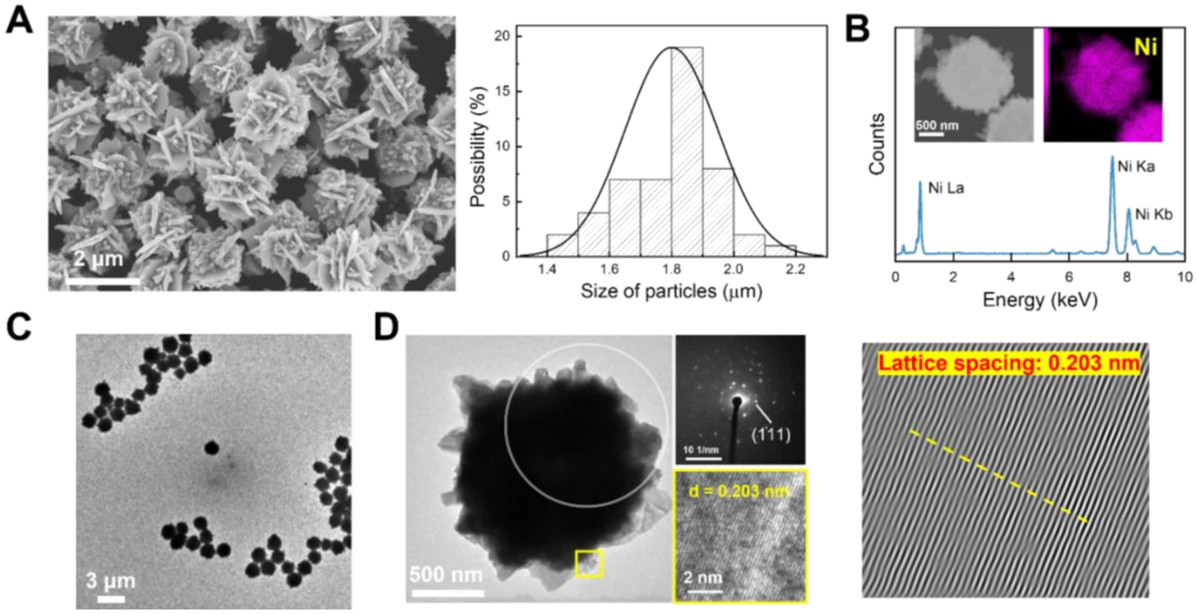

Figure S1. Characterization of ECFNCSs.

A, SEM, **B**, EDX, **C**, TEM and **D**, HRTEM of ECFNCs. SEM image shows the flower-like rough surface of ECFNCs and the quantitative statistics reveals the average diameters of ~1.8 μm. Energy-dispersive X-ray spectroscopy and elemental mapping confirm the nickel nature of the particles. Analysis of HRTEM result confirms that the ECFNCs are comprised of *fcc* nickel nanocrystals.

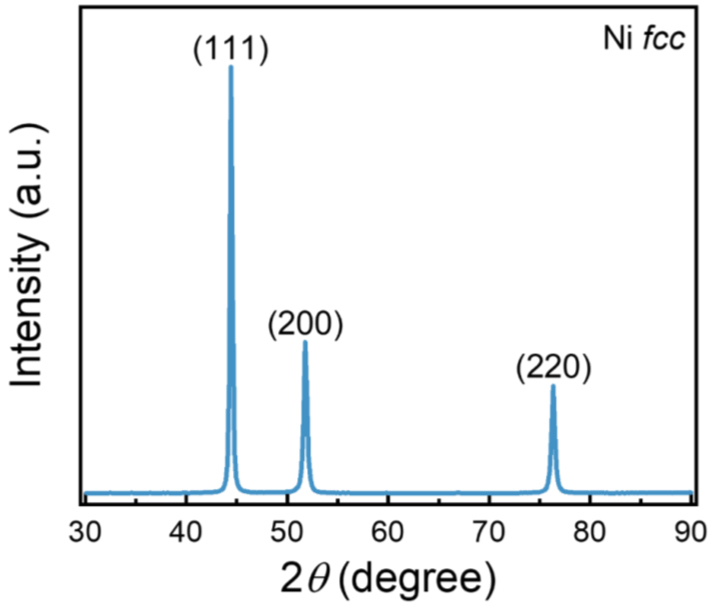

Figure S2. XRD characterization of ECFNCs.

X-ray diffraction unravels the *fcc* crystalline structure of ECFNCs.

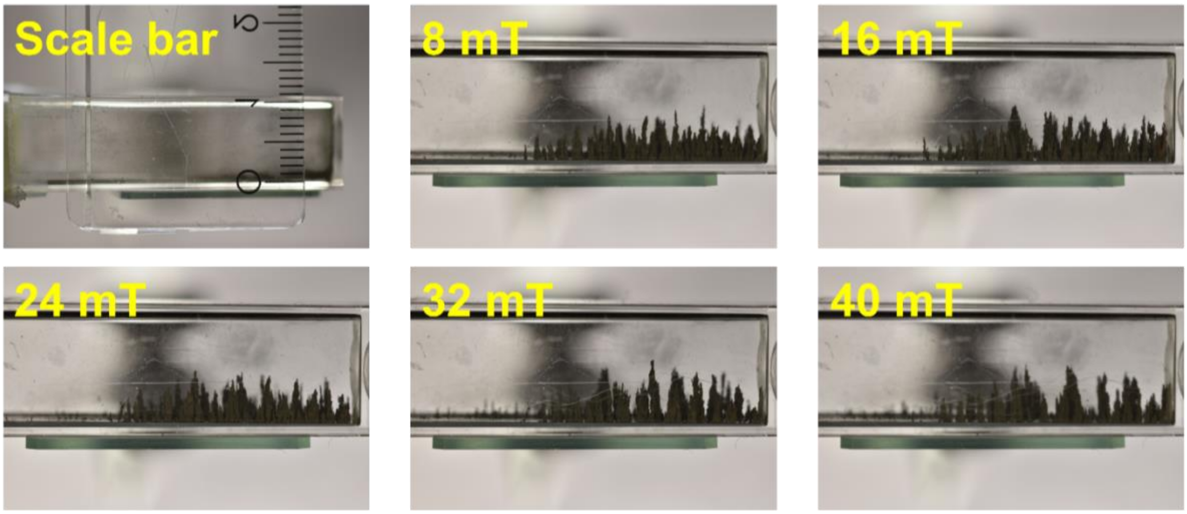

Figure S3. The unconstrained assembly of ECFNCs.

The side view images of pillars upon application of magnetic field *B*.

Table S1. The summarized details of unconstrained pillars at different magnetic fields.

B (mT)	Data 1		Data 2		Data 3		Data 4		Data 5		**Average**		
	No. of pillars	*H* (mm)	No. of pillars	*H* (mm)	No. of pillars	*H* (mm)	No. of pillars	*H* (mm)	No. of pillars	*H* (mm)	No. of pillars		*H* (mm)
8	36	2.765	29	2.823	29	2.791	31	2.647	27	2.706	30.4	**30**	**2.7464**
16	35	2.956	29	3.019	27	2.968	27	2.682	21	2.716	27.8	**28**	**2.8682**
24	32	3.030	24	3.246	26	3.051	26	3.028	20	2.958	25.6	**26**	**3.0626**
32	28	3.361	22	3.456	23	3.106	24	3.271	19	3.106	23.2	**23**	**3.2600**
40	23	3.537	23	3.629	20	3.464	24	3.347	18	3.630	21.6	**22**	**3.5214**

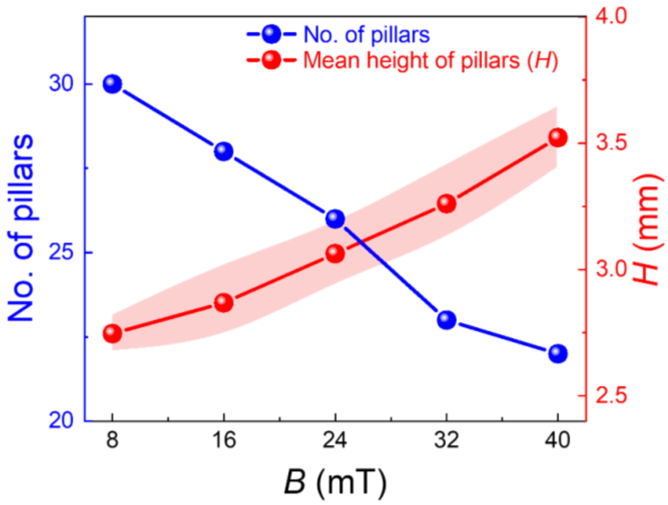

Figure S4. The height and population of unconstrained pillars as a function of *B*.

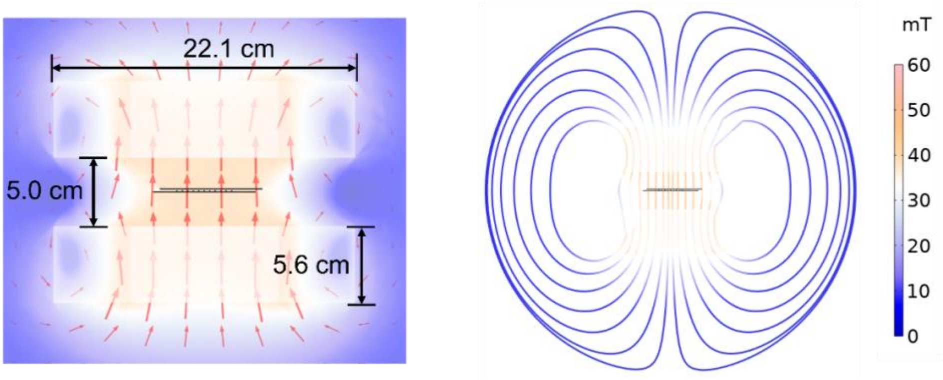

Figure S5. Simulated magnetic field profile.

The simulated magnetic fields generated by a pair of Helmholtz coils, and the magnified field profile around the sample site.

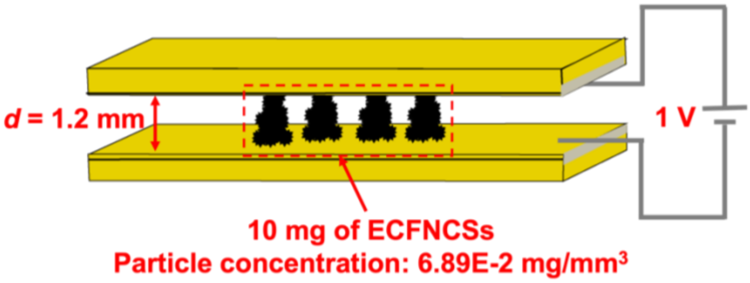

Figure S6. Schematic of the electric cell used in the experiment.

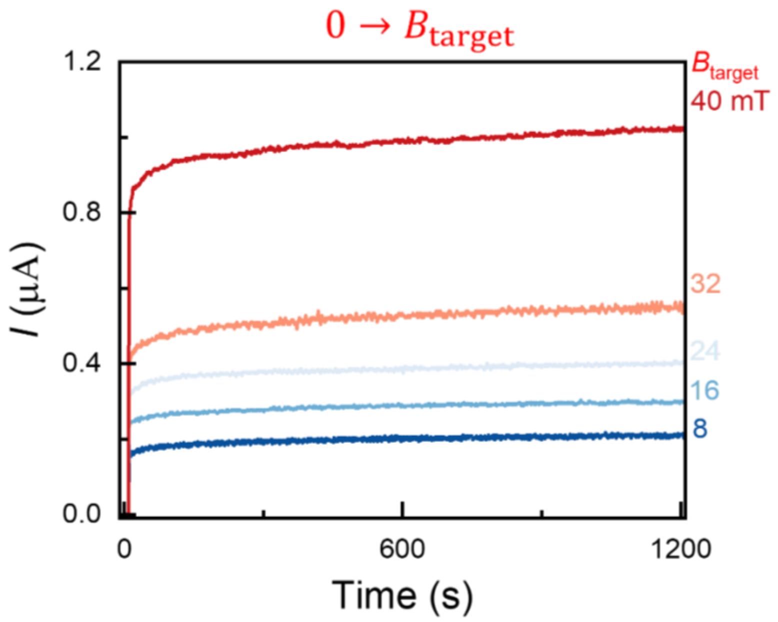

Figure S7. Electric performance during the process of 0 → B_{target}.

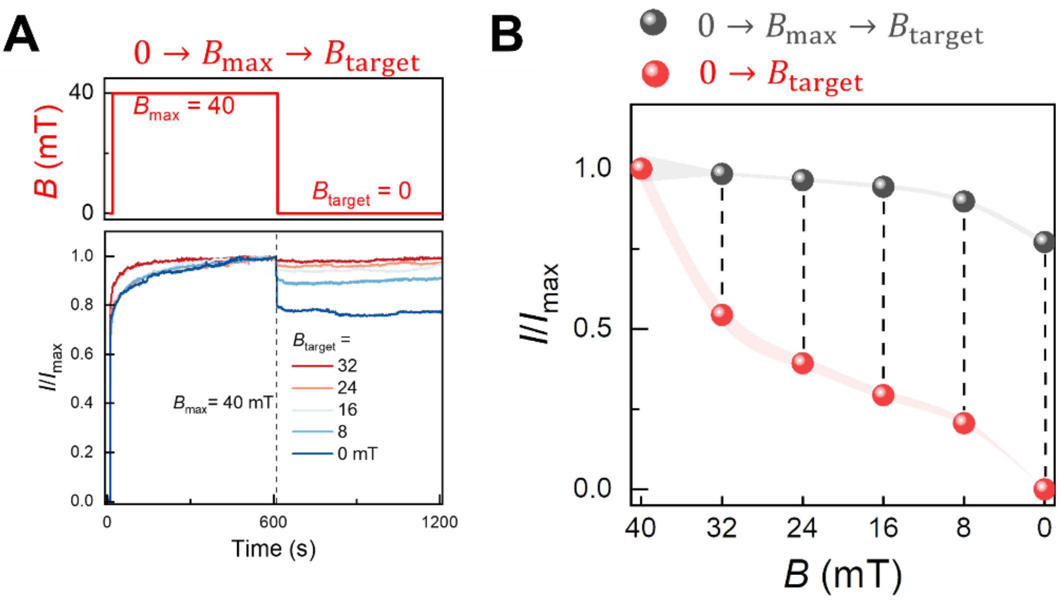

Figure S8. Suppressed current based memory.

A, Electric performance during the process of 0 → B_{max} → B_{target}. **B,** The disassembly of magnetic pillars by first applying B_{max} at 40 mT for 600 s and then decreasing it to B_{target} (0 → B_{max} → B_{target}, grey dots), in comparison to those (red dots) acquired using 0 → B_{target}.

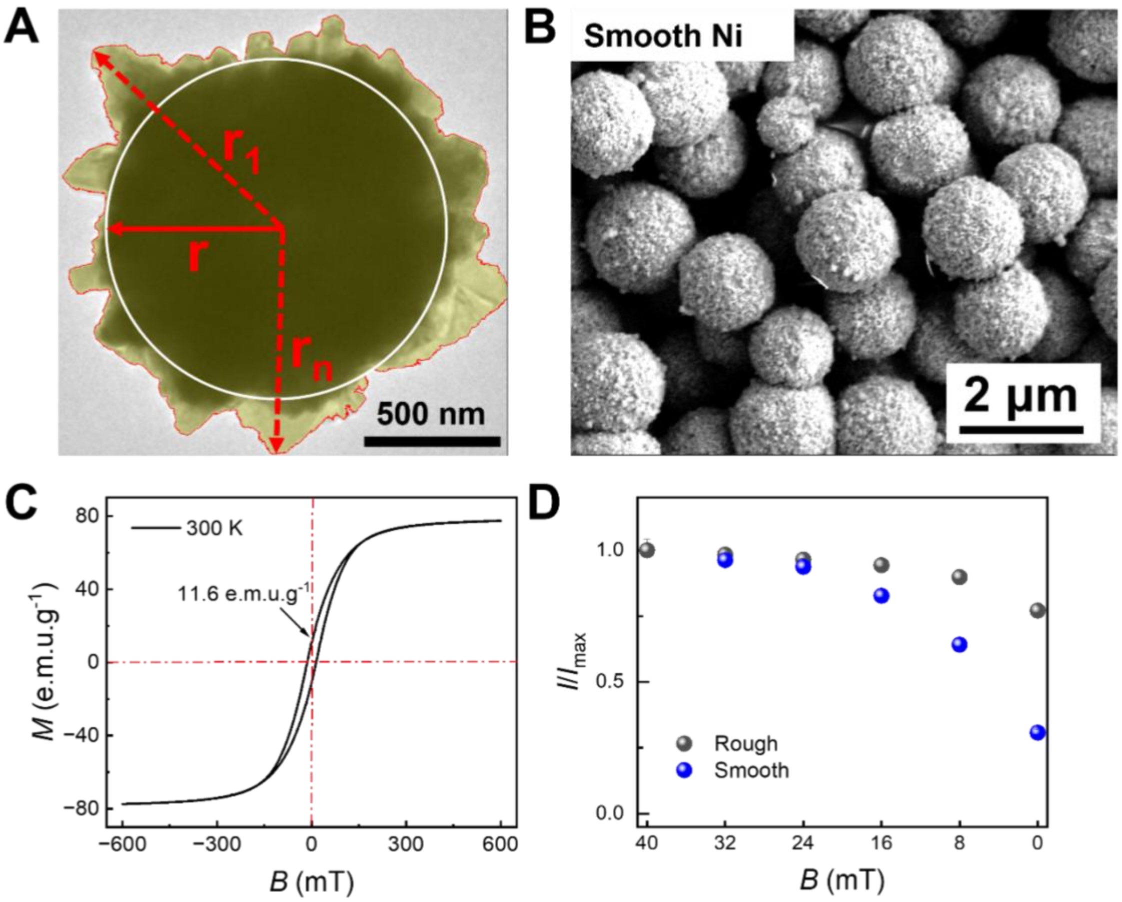

Figure S9. Characterizations of nickel particles.

A, The quantification of the particle's surface roughness. It simply defined by calculating the average value of r/r_n. **B,** SEM image of soft ferromagnetic Ni particles with smooth surface, their mean diameter is 1.6 μm. **C,** Magnetic moment *M* of smooth Ni particles as a function of *B*. **D,** The disassembly of rough particles ECFNCs and smooth Ni particles by first applying B_{max} at 40 mT for 600 s and then decreasing it to different B_{target}.

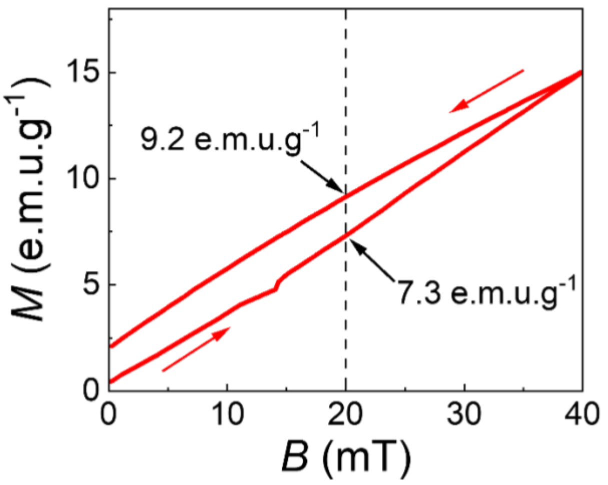

Figure S10. Mass magnetic moment *M* as a function of external magnetic *B*.

The *M* value measured after 0 → 40 mT → 20 mT process (9.2 e.m.u.g^{-1}) is higher than that of directly increasing *B* to 20 mT (7.3 e.m.u.g^{-1}).

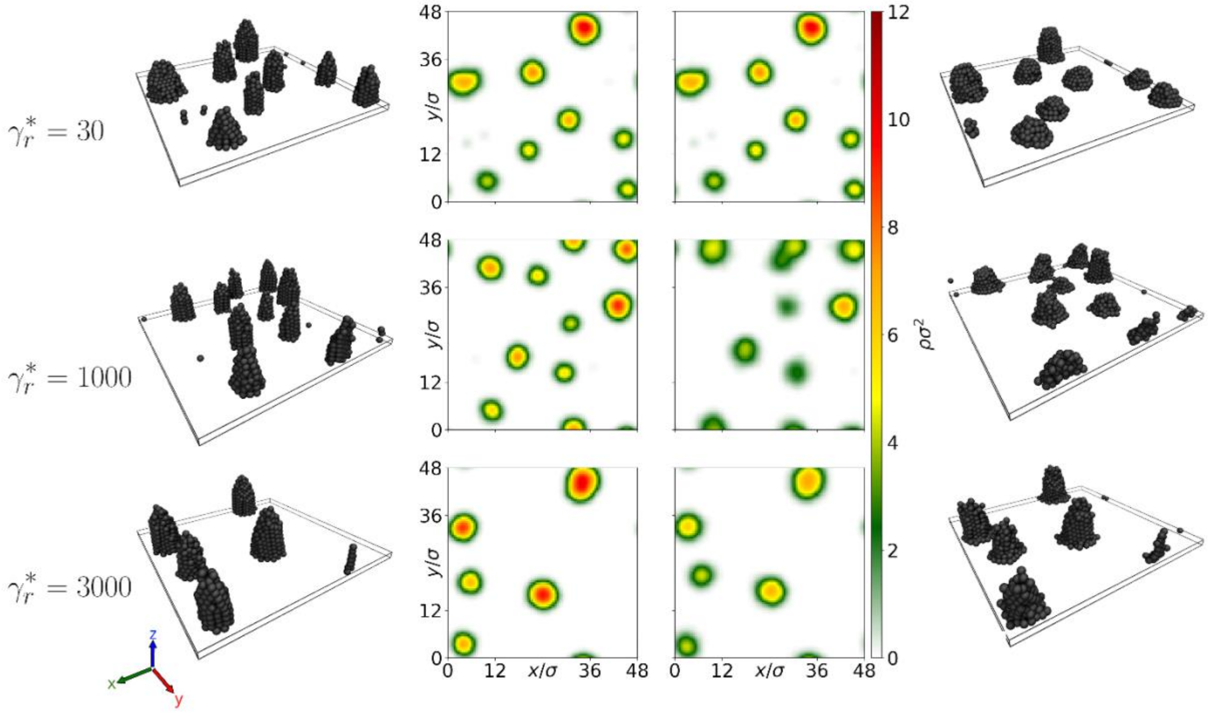

Figure S11. The effect of rotational friction γ_r^* on structural memory in the simulations. At left, visualizations and density profiles corresponding to main manuscript Fig. 3B at reduced simulation time 21 (at peak magnetization) and at right, the corresponding data at time 27 (after magnetic field retraction). Reduced simulation time is measured in units of 10^7 time steps. Magnetic field strength corresponds to Fig. 3B. Increasing the rotational friction between particles enhances the structural retention after the magnetic field is retracted. $\rho\sigma^2$ is two-dimensional dimensionless number density of particles.

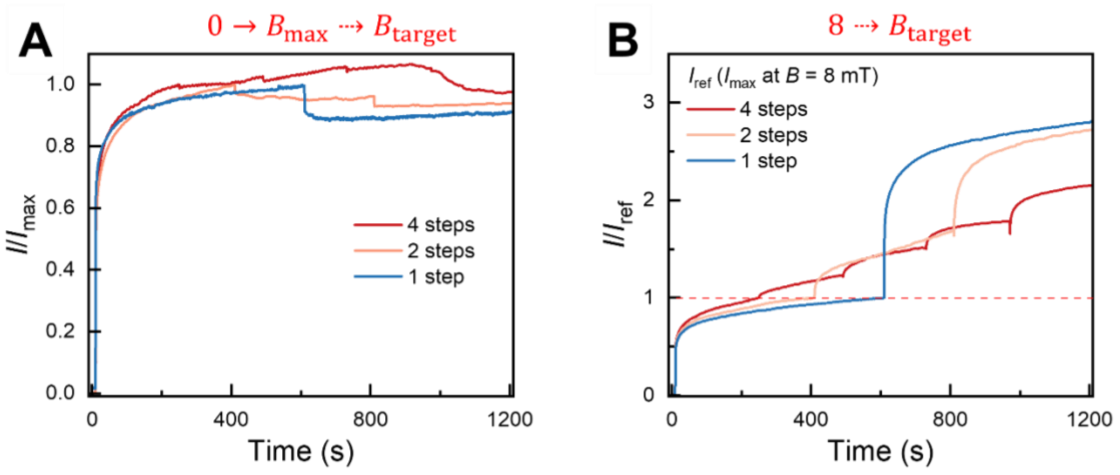

Figure S12. Suppressed current based on structural memory.

Electric characterization during the processes of (**A**) $0 \rightarrow B_{\mathrm{max}} \dashrightarrow B_{\mathrm{target}}$ and (**B**) $8 \dashrightarrow B_{\mathrm{target}}$.

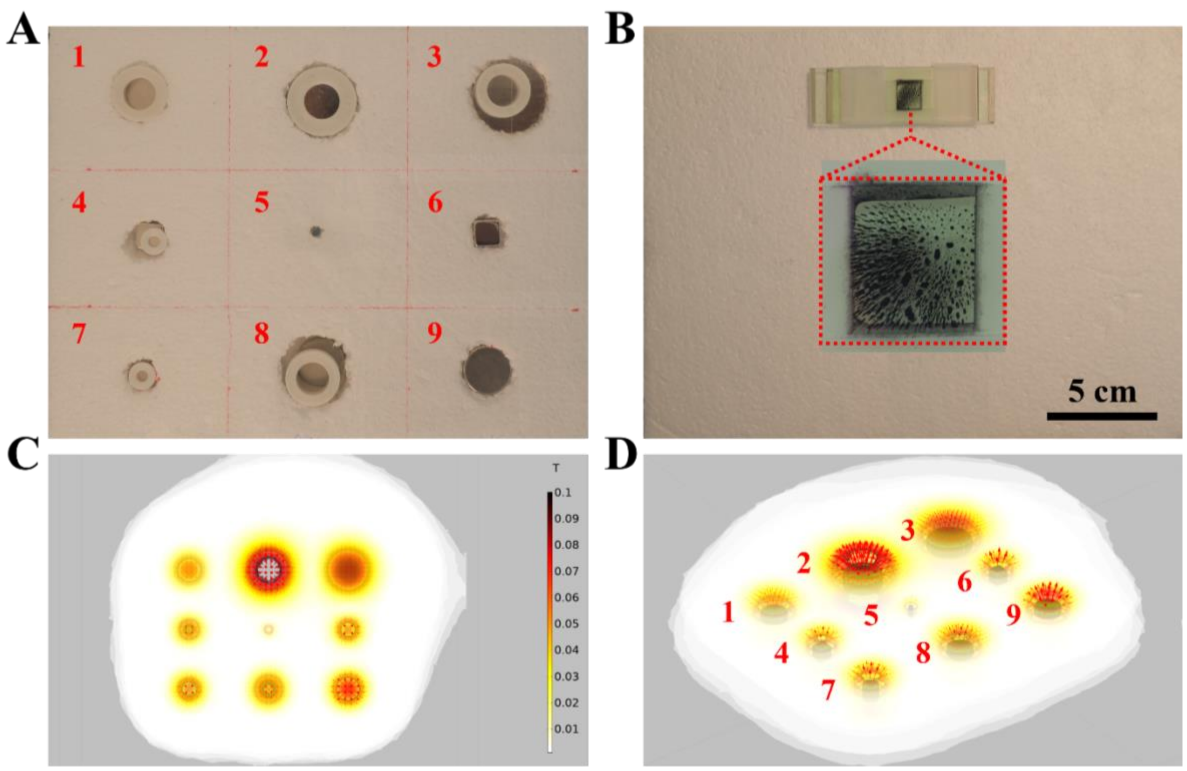

Figure S13. An artificial magnetic landscape.

A, Optical photographs of a man-made magnetic landscape comprising nine rubidium magnets in a 3×3 matrix, which are embedded in a foam board. **B**, The magnetic sensory receptor placed on the surface of the foam board. **C-D**, The simulated magnetic flux density landscape with COMSOL.

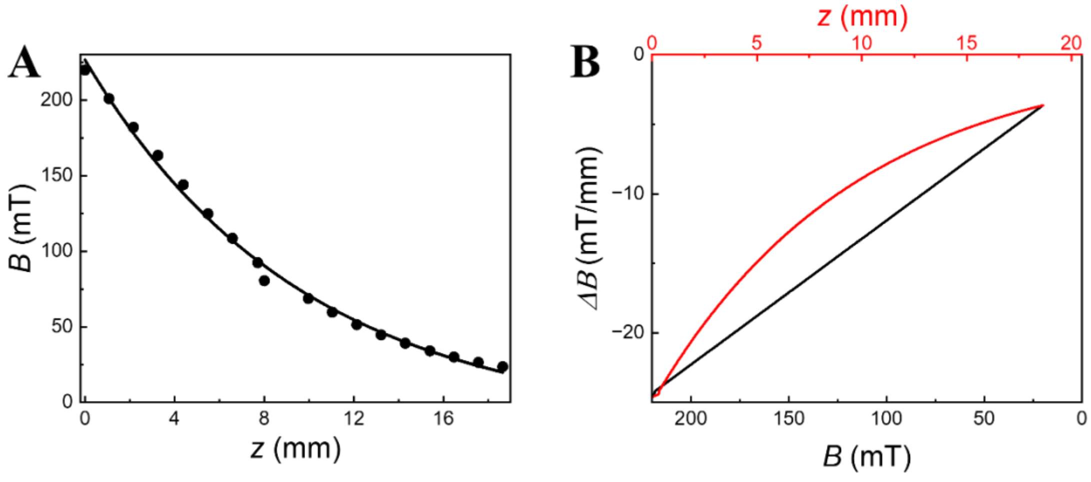

Figure S14. Magnetic fields of the typical disc magnet (magnet No. 8 and 9) used in experiments.

A, Magnetic field strength on the magnet axis as a function of distance from the surface of the magnet, showing experimentally measured data (dots) and theoretical fits (lines). **B**, The corresponding vertical field gradients $\Delta B = dB/dz$.

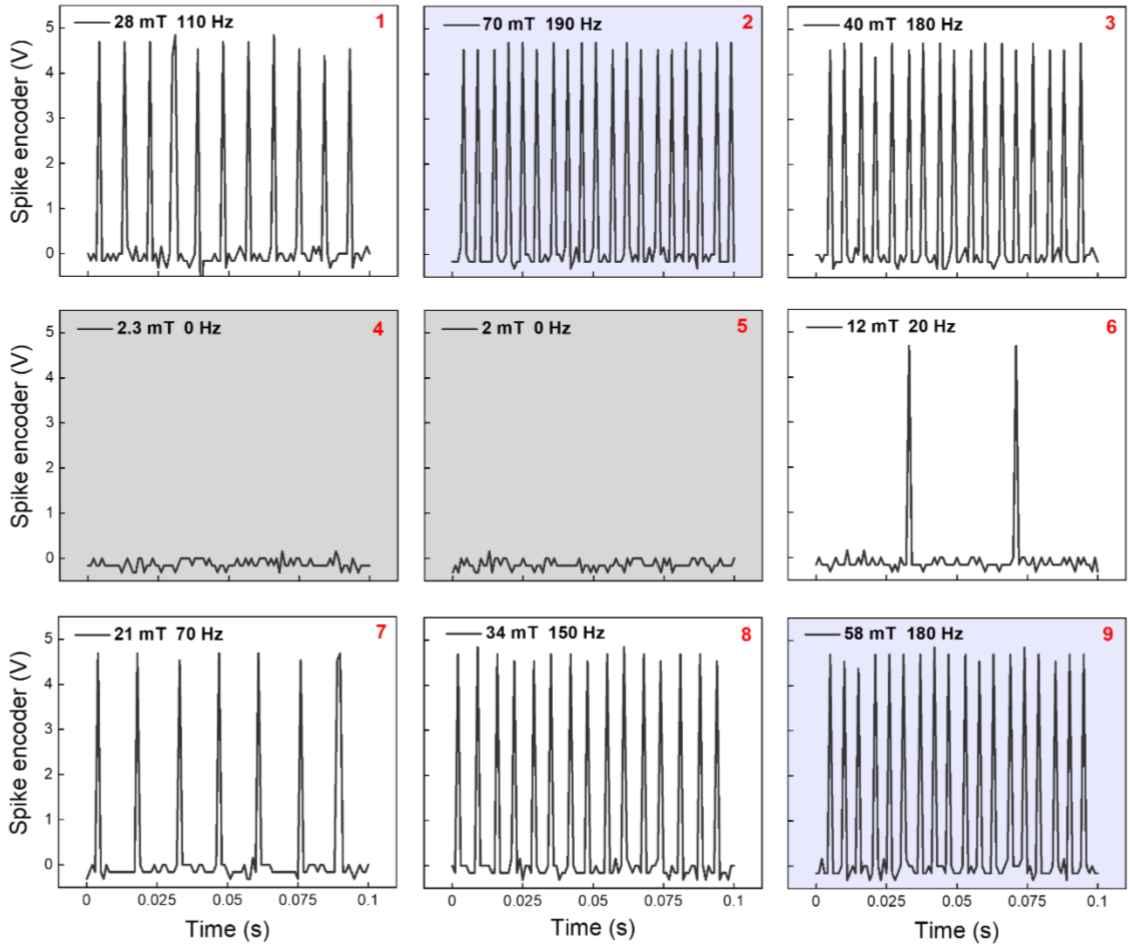

Figure S15. Mapping the magnetic landscape with the magnetic sensory system.

The fields at positions 4 and 5, and 2 and 9 cannot be perceived and discerned because they are beyond the lower and upper limits of the sensation, respectively.

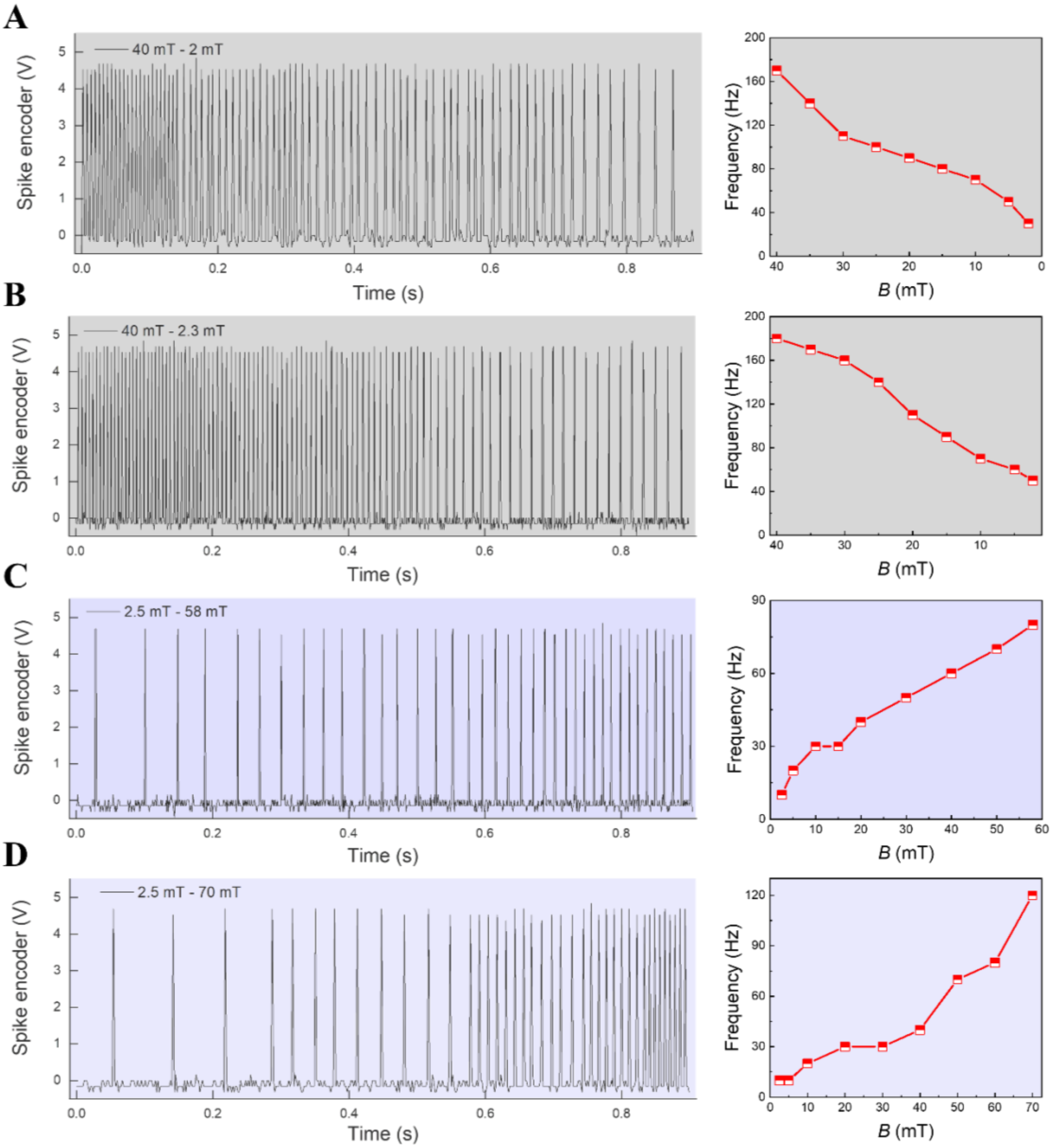

Figure S16. Magnetic sense training by promoted and depressed adaptation.

A-B, Promoting the sensory system by first approaching it to a high-strength magnetic field at 40 mT (dB/dz = 5.7 mT/mm) and then gradually to the aimed field at 2 (dB/dz = 1.7 mT/mm) (**A**) and 2.3 (dB/dz = 1.8 mT/mm) (**B**) mT, respectively. **C-D**, Depressing the sensory system by gradually approaching it to the aimed field from 2.5 (dB/dz = 1.8 mT/mm) to 58 (dB/dz = 7.6 mT/mm) (**C**) and 70 (dB/dz = 8.8 mT/mm) (**D**) mT, respectively.

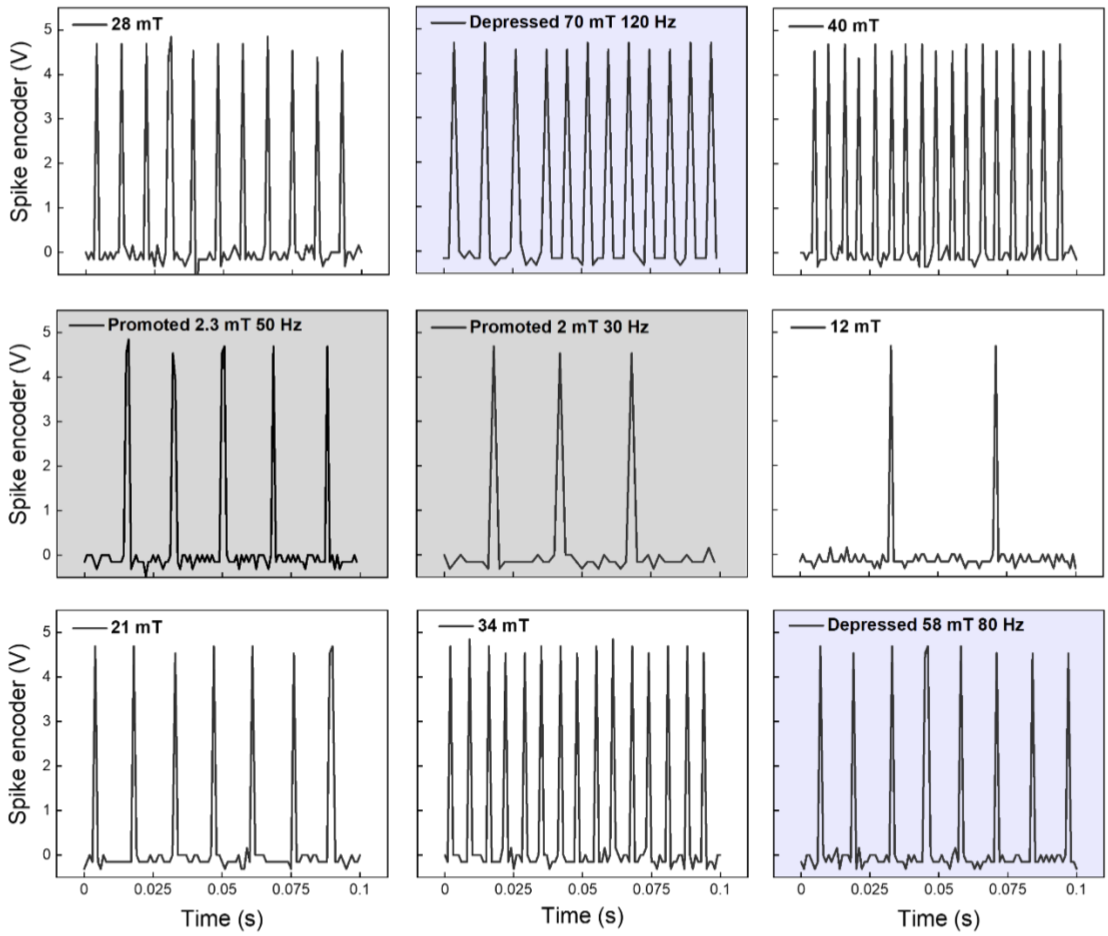

Figure S17. The restored mapping of the magnetic landscape after training.
