# Supplementary material for: Trainable bioinspired magnetic sensitivity adaptation using ferromagnetic colloidal assemblies
Source: Cell Rep Phys Sci. 2024 Apr 17;5(4):101923. doi: 10.1016/j.xcrp.2024.101923 (PMC11043831; doi:10.1016/j.xcrp.2024.101923)
Supplement: Document S2. Article plus supplemental information [file mmc2.pdf]

Cell Reports
Physical Science

CellPress
OPEN ACCESS

Article

Trainable bioinspired magnetic sensitivity adaptation using ferromagnetic colloidal assemblies

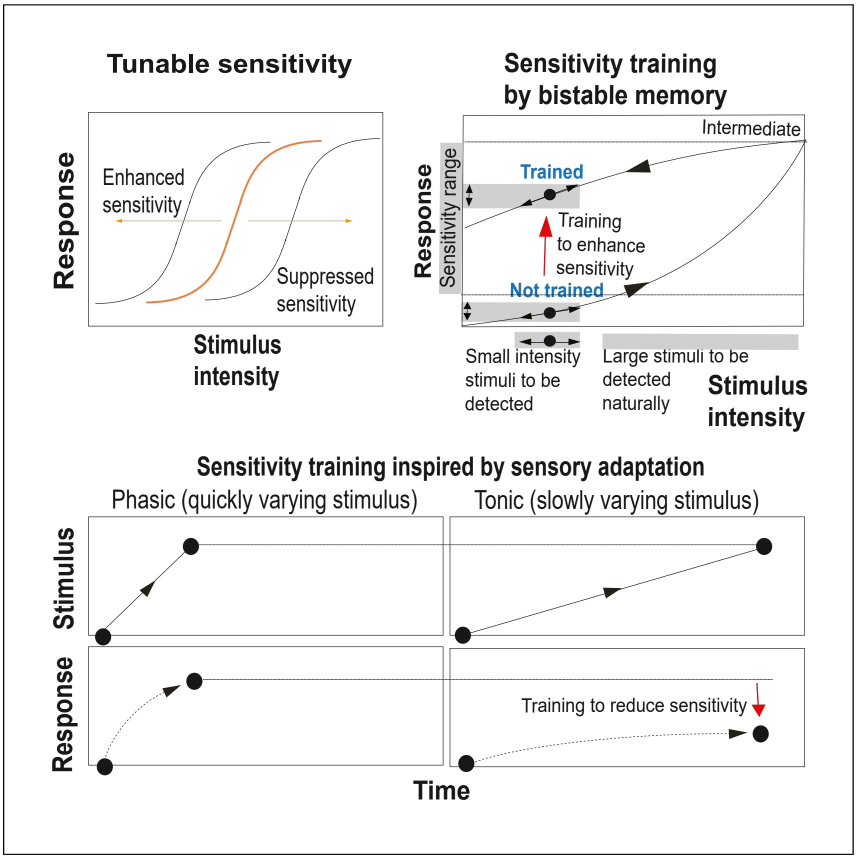

Memory is a trait typically associated with living organisms, distinct from inanimate matter. Liu et al. show structural memory in magnetic field-driven particle assembly, allowing adaptive sensation of magnetic fields upon signal transduction.

Xianhu Liu, Hongwei Tan, Emil Stråka, ..., Maria Sammalkorpi, Olli Ikkala, Bo Peng

olli.ikkala@aalto.fi (O.I.)
pengbo006@gmail.com (B.P.)

Highlights

Colloidal jamming leads to structural memory

Structural memory is also validated by Brownian dynamic simulations

Structural memory can be transduced into electric memory

A magnetic sensation system is devised based on structural memory

Liu et al., Cell Reports Physical Science 5, 101923
April 17, 2024 © 2024 The Authors. Published by Elsevier Inc.
https://doi.org/10.1016/j.xcrp.2024.101923

Cell Reports
Physical Science

CellPress
OPEN ACCESS

Article

Trainable bioinspired magnetic sensitivity adaptation using ferromagnetic colloidal assemblies

Xianhu Liu,[1] Hongwei Tan,[1] Emil Stråka,[2] Xichen Hu,[1] Min Chen,[3] Sebastiaan van Dijken,[1] Alberto Scacchi,[1,2,4] Maria Sammalkorpi,[2,4] Olli Ikkala,[1,*] and Bo Peng[1,3,5,*]

SUMMARY

Nature has already suggested bioinspired functions. Beyond them, adaptive and trainable functions could be the inspiration for novel responsive soft matter beyond the state-of-the-art classic static bioinspired, stimulus-responsive, and shape-memory materials. Here, we describe magnetic assembly/disassembly of electrically conducting soft ferromagnetic nickel colloidal particles into surface topographical pillars for bistable electrical trainable memories. They allow magnetic sensing with adaptable and rescalable sensitivity ranges, enabled by bistable memories and kinetic concepts inspired by biological sensory adaptations. Based on the soft ferromagnetism of the nanogranular composition and the resulting rough particle surfaces prepared via a solvothermal synthesis, triggerable structural memory is achieved by the magnetic field-driven particle assembly and disassembly, promoted by interparticle jamming. Electrical conversion from current to frequency for electrical spikes facilitates rescalable and trainable frequency-based sensitivity on magnetic fields. This work suggests an avenue for designing trainable and adaptable life-inspired materials, for example, for soft robotics and interactive autonomous devices.

INTRODUCTION

Over the past decades, bioinspiration has facilitated numerous approaches for materials science, ranging from nanocomposites with synergistic combinations of strength and toughness, superwetting surfaces, and structural colors, to adhesion, among others.[1–7] Such functional properties have typically been static (i.e., being in energy equilibrium or kinetically trapped). They have been achieved by self-assemblies, structural hierarchies,[8] and using carefully balanced chemical and physical interactions. Therein also, bioderived materials have increasingly been used as components, aiming at promoted sustainability, for example, by using modified silks, amyloids, nanocelluloses, and concepts allowed by synthetic biology.[8–10]

However, a growing quest for more dynamically adaptive multifunctional materials is foreseen toward "materials intelligence" or "physical intelligence," aiming at new concepts for, as examples, future soft robots and interactive materials, ultimately toward interfacing with biomedical applications.[11–13] Thus, biological behaviorism[14] and psychophysical behaviors[15,16] could inspire the next generation of adaptive responses for interactive materials properties,[17,18] beyond the classic static bioinspired properties. The aimed-for behaviors could be preprogrammable within

[1]Department of Applied Physics, Aalto University, P.O. Box 15100, 00076 Aalto, Finland

[2]Department of Chemistry and Materials Science, Aalto University, P.O. Box 16100, 00076 Aalto, Finland

[3]Department of Materials Science, Advanced Coatings Research Center of Ministry of Education of China, Fudan University, Shanghai 200433, China

[4]Department of Bioproducts and Biosystems, Aalto University, P.O. Box 16100, 00076 Aalto, Finland

[5]Lead contact

*Correspondence: olli.ikkala@aalto.fi (O.I.), pengbo006@gmail.com (B.P.)

https://doi.org/10.1016/j.xcrp.2024.101923

 © 2024 The Authors. Published by Elsevier Inc.
This is an open access article under the CC BY license (http://creativecommons.org/licenses/by/4.0/).

the material structures to facilitate designed sequences of responses based on selected stimuli. Aiming at such materials to respond as if they would have a glimpse of seeming intelligence, it would be natural to seek inspiration from the biological behaviors from adaptation, learning, and cognition.[14–16,19] Habituation and sensitization[14] are among the simplest unsupervised learning processes showing suppressed or enhanced responses upon repeated stimuli. However, sensory adaptation[15,16,19] typically addresses responses for continuous stimuli, which can be slowly varying (tonic) or quickly varying (phasic) to adjust and rescale the sensitivity to the varying stimulus forms and intensities; classical conditioning[13] addresses elementary supervised learning processes upon association between two stimuli to achieve a new stimulus response.

Characteristic of all such behavioral functions is that they require triggerable memories—in other words, they are not reversible upon application and removal of the stimuli, unlike the classic stimulus response and shape-memory materials, which is challenging to realize using artificial systems. Still, some of them have already inspired new simplistic functional materials using classical conditioning, response plasticity, and spiking.[20–22] The simplest form of triggerable memory is bistability (i.e., difference between increase and decrease of the applied stimulus, so far amply described in neuromorphic devices in current-voltage base).[23]

Note that such materials would be conceptually different from the classic stimulus responsive[24–26] or shape-memory[27,28] materials. Such classical synthetic material responses are typically limited to fixed energy equilibria or kinetic trapped states, showing reversibility under repeated stimulus exposures (i.e., they do not evolve to new responses or functions). Also, typically, they show no adjustments or adaptations to different strengths of the stimuli.[25,29]

Among the different stimuli, the magnetic field is a relevant stimulus for emerging interactive devices and soft robots because it can be applied and sensed remotely.[30] Intriguingly, in biology, the adaptive magnetic sensing allows animals to navigate under magnetic field gradients and changes, based on magnetic particle (magnetosome) responses.[31] They allow response rescaling and adjustment to detect magnetic field changes under different environments.[32]

Differing from classical man-made sensors with fixed resolution limits aiming to detect absolute values, biological sensing typically aims at the detection of stimulus changes using rescalable adaptive dynamic ranges, denoted as biological sensory adaptation.[15,16] Here, we show trainable sensory concepts inspired by bistable memories and biological sensory adaptation involving electrical responses upon different levels of magnetic field stimuli (Figure 1). Anisotropic one-dimensionally extended pillared surface topographies are selected because hairy surfaces are ubiquitous to facilitate a multitude of biological functions, from debris removal in mucus, thermal control, hearing, wetting control, to sensing and actuation.[3] As a versatile model system, we use electrically conductive soft ferromagnetic nickel colloidal particles (ECFNCs) as model materials for on-demand dynamic pillared assemblies because they allow the combination of electrical responses to the magnetically driving assembly, as described by us recently in another context.[22] Here, we show how they can be used as magnetic field sensors with trainable and adaptive sensing upon connecting to ring-oscillator electrical circuits to generate magnetic field-dependent electrical spiking based on the sensed field strengths.

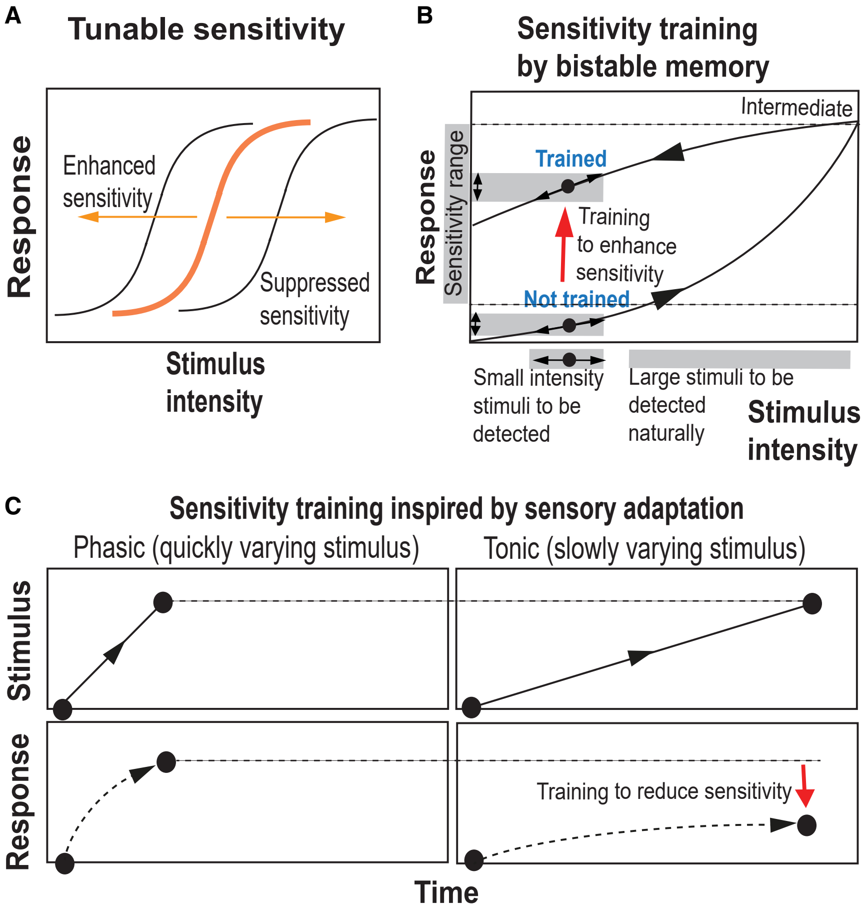

Figure 1. Illustrations for trainable responses allowed by bistable memories and kinetics of stimulus applications, different from the classical stimulus-responsive and shape-memory materials

(A) The sensitivity of adaptations can lead to the enhancement or suppression of the responses to detect stimulus changes at a larger dynamic range.

(B) Sensitivity training by bistable memory to allow improved sensitivity.

(C) Sensitivity training by sensory adaptation inspired by psychophysics involving slowly (tonic) or quickly (phasic) stimuli, incorporating a memory, to allow suppressed sensitivity.[15,16]

RESULTS

Synthesis and assembly of ECFNCs

The ECFNCs are synthesized via a solvothermal approach.[22,33] The precursor $NiCl_2$ is reduced to nickel colloids by ethylene glycol at 200°C in the presence of NaOH (see experimental procedures). The ECFNCs are constituted by randomly interlaced nanoflakes with a mean overall diameter of 1.8 μm, and they show a narrow polydispersity of 8.2%, as shown in the representative scanning and transmission electron microscopy (SEM, TEM) images (Figure S1). These results are corroborated by energy-dispersive X-ray spectroscopy (EDX) and elemental mapping (Figure S1B). The selected-area electron diffraction reveals the polycrystalline nature of the ECFNCs, comprising *fcc* nickel nanograins (Figure S1D, insets). The mean size of the nanograin is 35.4 nm, as estimated from X-ray diffraction (XRD) patterns (Figure S2). The ECFNCs are soft ferromagnetic at 300 K, showing small coercivity B_c = 13.9 mT and remanence M_r = 7.4 electromagnetic units (e.m.u.)/g, respectively (Figure 2A), as also verified by the field cooling and zero-field-cooling (ZFC) measurements (Figure 2B).[33,34]

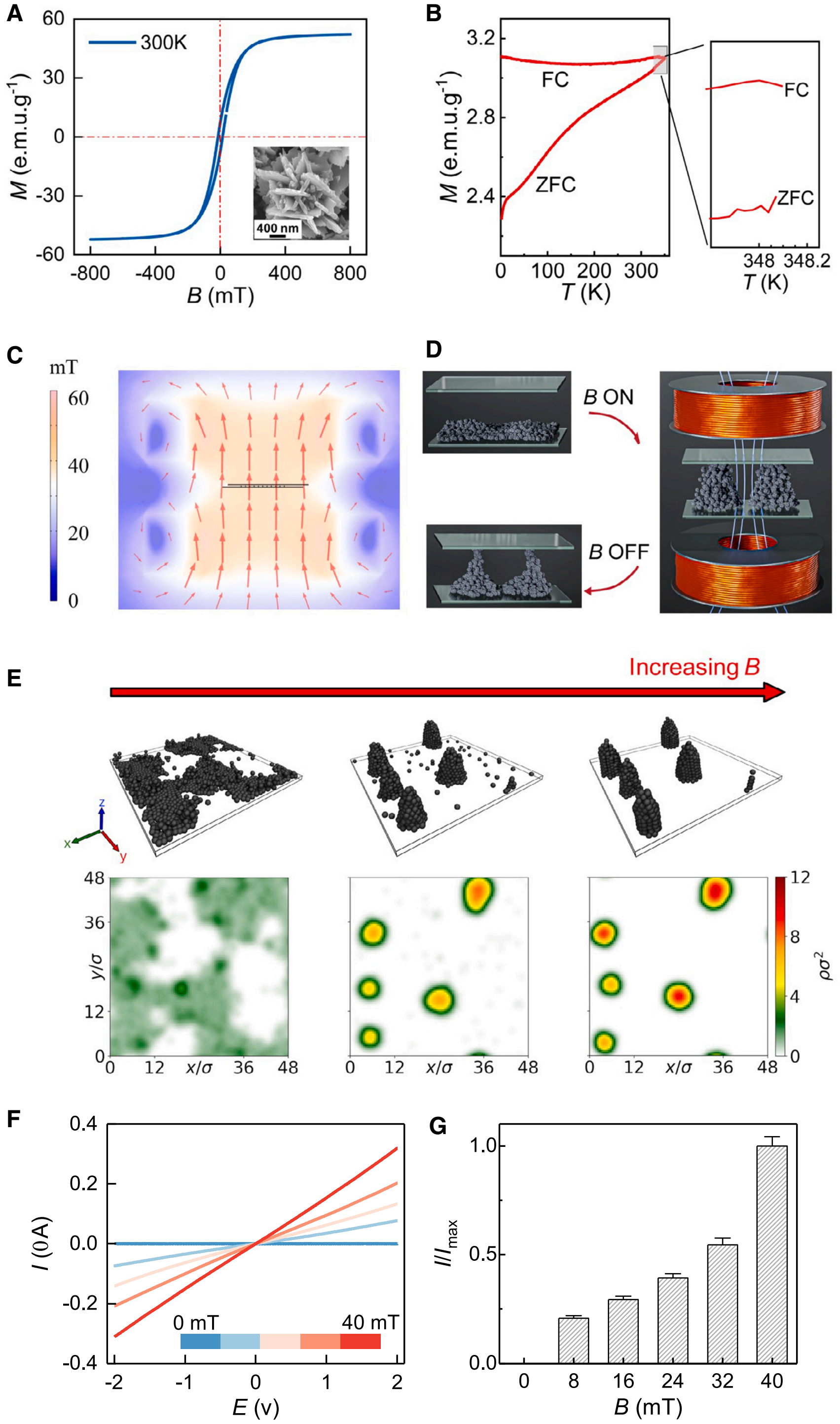

Figure 2. Assembly of ECFNCs

(A) Mass magnetization *M* of ECFNCs as a function of the exposed magnetic field *B*.

(B) The FC and ZFC measurements.

(C) The simulated magnetic field lines at the site of the sensor, allowed by an electromagnet. The simulation was conducted using COMSOL Multiphysics version 5.5.

(D) Scheme for the magnetic field-driven pillared assemblies, disassemblies, and structural jamming of ECFNCs.

Figure 2. *Continued*

(E) Visualizations and the corresponding number density profiles of pillars formed in Brownian dynamics simulations with increasing B (for experimental data, see Figures S3 and S4). $\rho\sigma^2$ is the 2-dimensional dimensionless number density of particles.

(F) Linear sweep voltammetry verifies the B-dependent electrical conductivity of the ECFNCs. The voltages for linear sweep range from −2 to 2 V, with a step size of 5 mV.

(G) Stepwise magnetic field increases in a fraction of seconds lead to increases in the electric conductivities upon the formation of assembled ECFNC pillars. T-shaped error bar overlays the bar chart.

Such soft ferromagnetic ECFNCs are fundamentally interesting because they have small coercivity and thus small mutual magnetic interactions at zero exposed field despite their micrometer size facilitated by their nanogranular structure, unlike classical hard ferromagnetic nickel particles of such sizes. Therefore, ECFNCs are useful model materials to explore assemblies using competing magnetic and gravitational sedimentation effects. Upon the application of magnetic field B (Figure 2C), the ECFNCs form pillars (Figures 2D and S3) whose heights increase as a function of B on a substrate (Figure S4; Table S1), having approximately conical shapes upon competing magnetic and gravitational effects involving jamming.[22] To achieve further understanding for the pillar formation, Brownian dynamics simulations with model interparticle interactions were performed to account for the ECFNC characteristics and the effect of the magnetic field B. Brownian dynamics is a particle-based simulation approach that captures the effects of surrounding medium, interparticle interactions, and external forces on the assembly. The results indicate that at low B, the particles aggregate on the substrate, with large base area island formation (Figure 2E). As B increases, the particles begin to assemble into separate approximately conically shaped pillars, with pillar height increasing with B (Figure 2E).

Next, to demonstrate magnetic field sensing, 10-mg ECFNCs are confined between two electrically conducting indium tin oxide (ITO)-coated glass slides that are separated by 1.2 mm, leading to an average particle concentration of 6.9×10^{-2} mg/mm^3 within the confined volume. Such an element is wired to form an electrical circuit driven by a constant voltage V (Figures S5 and S6). Upon application of a homogeneous magnetic field B by an electromagnet (Figure 2C) opposite to the gravity, the particles assemble to electrically conducting pillars, schematically shown in Figure 2D, thus providing magnetically tunable electrical conduction between the two electrodes (Figure 2F).[22,35] Due to the confinement, the pillars adopt approximately truncated conical shapes.[22] Increasing B leads to promoted accumulation of particles to laterally thicker truncated cones,[22] thus increasing the available current I upon constant V, until the maximum current I_{max} is achieved for the maximum available magnetic field 40 mT (Figure S7). Thus, we analyze the increasing relative value I/I_{max} as a function of increasing applied B, shown in Figure 2G.

Structural memory and kinetics of the pillar assembly and disassembly

Next, the history dependence on the magnetic field effect on the pillar assembly and disassembly is explored, manifesting in the electrical conductivity. Figure 3A shows that initially at $B = 0$, there is vanishing electric current due to the absence of pillars, whereas a single stepwise increase of B up to 40 mT in a fraction of a second leads to $I/I_{max} = 1$ after stabilization during 600 s due to pillar assembly. A subsequent single step removal of B leads to residual current $I/I_{max} = 0.77$, due to the incomplete disassembly of the pillars (schematically illustrated in Figure 2E). An asymmetry between the pillar assembly and disassembly manifests in the conductivity as a bistable electrical memory (Figure S8). An obvious mechanism therein is suggested by the

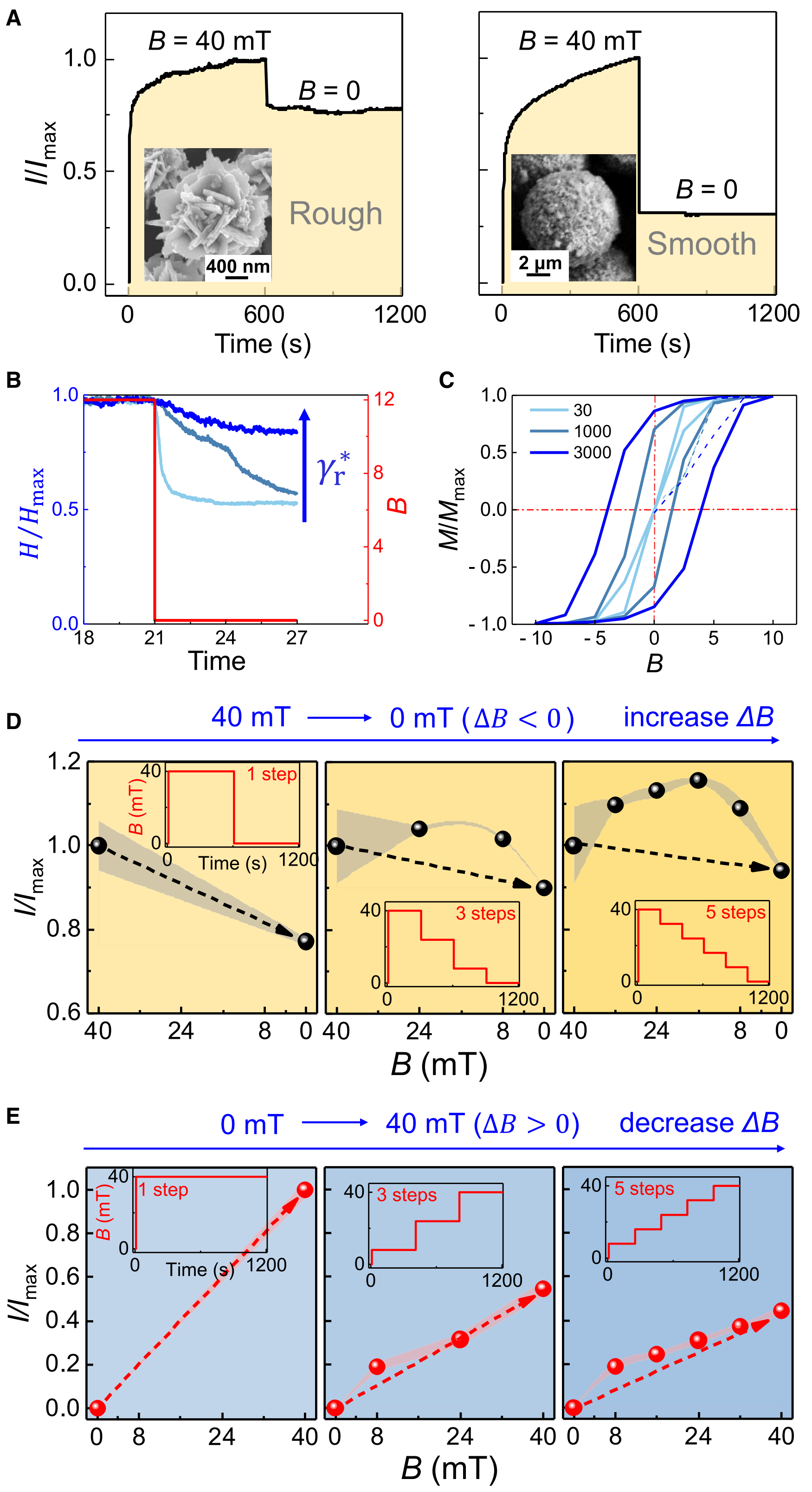
A
B = 40 mT
B = 0
Rough
400 nm
Smooth
2 μm
I/I_max
Time (s)
B
H/H_max
B
γ_r*
Time
C
30
1000
3000
M/M_max
B
D
40 mT ⟶ 0 mT (ΔB < 0) increase ΔB
1 step
3 steps
5 steps
B (mT)
Time (s)
I/I_max
B (mT)
E
0 mT ⟶ 40 mT (ΔB > 0) decrease ΔB
1 step
3 steps
5 steps
I/I_max
B (mT)

Figure 3. Structural memory and kinetic responses of the magnetic colloidal assembly and disassembly inspired by sensory adaptation

(A) Triggerable memory in the magnetically driven electrical circuit promoted by particles with rough and smooth surfaces upon first assembling pillars at B_{max} = 40 mT (defining I_{max}), followed by a single-step switching to B = 0.

(B) Normalized height H of the pillars in the Brownian dynamics simulations as a function of the particle friction coefficient γ_r^*. Time is measured in units of 10^7 time steps. The height of the pillars gives a qualitative indication of the conductivity, as upon in the constrained case the upper surface area defines the conductivity.

(C) Magnetization curves corresponding to the supracolloidal assembly of particles with different friction coefficient γ_r^* in the simulations.

(D) The residual currents upon first assembling the ECFNC pillars at B_{max} = 40 mT (defining I_{max}), followed by a single-step switching to B = 0 or a slow stepwise decrease in the field to 0 mT via 1, 3, and 5 steps during 1,200 s. The shaded area is the error range.

(E) The resulting I/I_{max} after assembling the pillars at 40 mT using 1, 3, or 5 steps. The shaded area in is the error range.

remanence magnetic interactions between the soft ferromagnetic ECFNC microparticles. Importantly, the surface roughness is also expected to contribute to the assembly/disassembly process because the reassemblies of the pillars can depend on the interparticle friction and jamming upon repackings of the pillars toward lower fields. We next explored by experiments and by simulation the effect of surface roughness. Experimentally, the surface roughness of the ECFNC particles (Figure 3A, left, inset) can be quantified by the averaged value of the maximum/minimum particle radius,[36,37] as depicted in Figure S9A, thus yielding a value of 1.27. Particles with smoother surfaces and corresponding micrometer sizes were synthesized using a related solvothermal approach (see experimental procedures and its subsection simulations), having a considerably smaller surface roughness of 1.01 (Figures 3A, right, inset, and S9B). Also, they were first exposed to 40 mT, and the field was subsequently removed in a single step, thus leading to considerably smaller residual current I/I_{max} = 0.31. This qualitatively pinpoints the essential effect of the surface roughness on the bistability due to surface friction and jamming. Notably, the remanences were roughly similar for the rough (7.4 e.m.u. g^{-1}) and smooth (11.6 e.m.u. g^{-1}) particles, respectively (Figures 2A and S10). That the remanence of the smooth particles is even slightly higher than that of rough particles excludes the conclusion that the assembly/disassembly anisotropy would be solely induced by magnetic remanence interactions. Therefore, both surface roughness and dipolar interactions contribute to the structural and electrical bistability. Their contributions can be illustrated using different residual fields, where the magnetic interactions progressively increase as a function of the residual B. Figures S8B and S9D show that after first exposing them to 40 mT, subsequently reducing the field to 32 and 24 mT in one step, both rough and smooth particles show high current retention of the pillars, whereas at smaller final field values, the smooth particles show drastically smaller residual currents. This qualitatively suggests that at high residual field values, the magnetic dipolar interactions dominate in the pillar assembly/disassembly and bistability, whereas at low residual fields the particle roughness and surface friction start to allow substantial contribution.

To allow a better understanding of the structural memory and the effect of surface roughness and interparticle friction, we conducted Brownian dynamics simulations of the ECFNC particle system with model interactions. The modeling involved fixed size particles with permanent magnetic dipoles, and we introduced the effect of surface roughness of the magnetic particles by varying the rotational friction parameter γ_r^* between the particles (see details in experimental procedures and its subsection simulations).[38] This simplified model description targets extracting the dependency

of roughness, magnetic moments, and external field to the retention of assembly in the system. Although a low friction surface cannot sustain the pillar structure at zero residual field after first being exposed to high field, a promoted higher friction parameter can lead to structural retention after retracting the magnetic field (Figures 3B and S11). Therefore, structural memory can be linked also to the surface roughness of the particles suggested both by simulation and experiments. In general, increasing the roughness enhances the structural memory, unraveling the vital role of the rough surface toward colloidal jamming and memory. Interestingly, the simulations also suggested tunable magnetic remanence for pillars based on the supracolloidal assemblies/disassemblies retention response (Figure 3C). The overall shape of the magnetization curves suggests that the "seeming" coercivity would increase as a function of the surface friction. This suggests that the friction provides a supracolloidal control for the promotion and hysteresis of the magnetic response.

Next, we turn to the kinetics of the pillar assemblies/disassemblies, inspired by biological psychophysical sensory adaptations involving so-called tonic and phasic stimuli (i.e., involving slowly and rapidly changing stimuli).[16] Here, we explore the effect of slow and quick magnetic field stepwise ramping on the conductivity. The disassembly of magnetic pillars is explored by first exposing B_{max} = 40 mT over 600 s to allow pillar stabilization, and subsequently the field is ramped to zero using one step (already shown in Figure 3A), next compared to 3- and 5-step ramping for 1,200 s (Figures 3D and S12A). The relative residual current (I/I_{max}) is higher when using several steps (i.e., a slower [tonic] B decrease. This is algorithmically similar to sensory adaptation as tonic responses. By contrast, upon increasing the magnetic field (stimuli) from 0 to 40 mT, the ECFNCs assemble into pillars, where a slow (tonic) increase leads to reduced electrical response in comparison to directly applying 40 mT in one step (Figures 3E and S12B). This is algorithmically inspired by the sensory adaptation as phasic responses.[15,16,39]

Trainable magnetic sensation systems in current and frequency base

Finally, we show that the present system allows trainable sensitivity and adaptive rescaling of the sensory ranges, allowing the detection of stimulus changes at a widely varying dynamic range of the stimuli. This is expected to be useful for the emerging soft robotics and autonomous interactive materials to detect changes in the environmental conditions under large dynamic ranges to adapt their responses. Inspiration is provided by biological sensory systems,[32] typically allowing us to sense stimuli/environmental changes sooner than their absolute values, such as in magnetoreception by a geomagnetic sensing in living systems to detect the magnetic field gradients in biological homing.[40] In this way, the approach is different from classical Gauss meters, where, for example, the Hall effect is a working principle for detecting the exact magnetic field values.[41] Promoted sensitivity training is shown here based on the current responses of the systems. Figure 4A illustrates the case in which an originally small field is first applied that does not allow pillar formation and detectable electrical response. In this case, the system can be trained toward an increased sensitivity by applying an intermediate high field to form the pillars and then to reduce the field back to the original field to be recorded. Due to the bistable memory, the process now allows the detection of the field value (and the changes nearby, see Figure 4A). However, if the original field to be recorded is excessively high, a reduced sensitivity is acquired by switching on the required field very slowly, facilitated by the kinetic memory of the present soft particle pillared structures—in other words, inspired by the tonic biological responses (Figure 4B), whereupon the responses and changes therein can be subsequently detected.

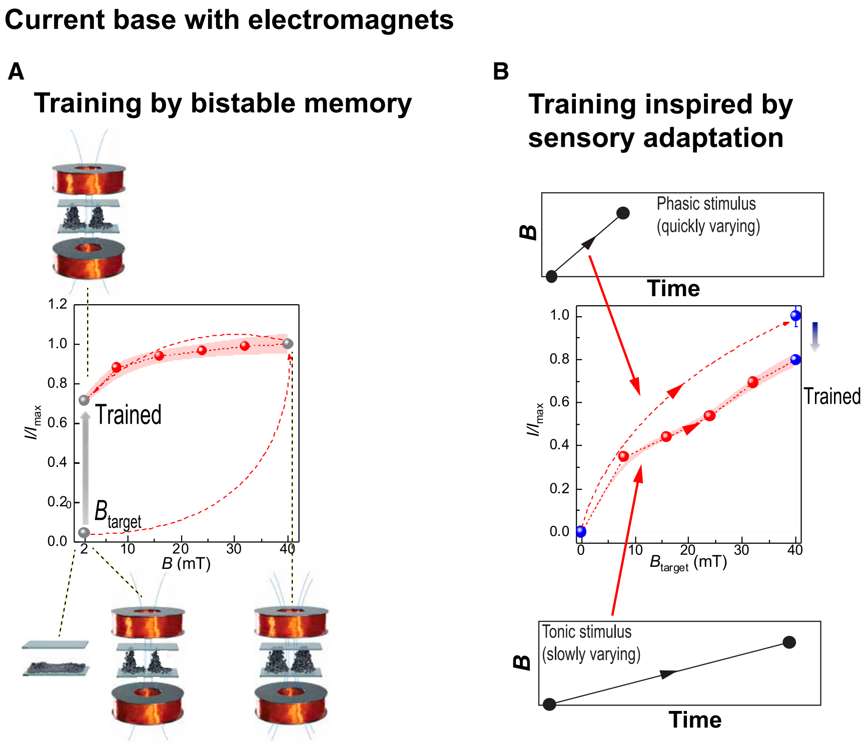

Figure 4. Magnetic field sensitivity training in the current base, explored using electromagnets
(A) Sensitivity training by bistable memory to promote the sensitivity, upon using high intermediate magnetic fields. In the bottom row of the insets from left to right, no pillars form at $B = 0$ mT, leading to $I/I_{max} = 0$. Upon applying a homogeneous magnetic field at $B = 2$ mT by a pair of electromagnets, the particles assemble into small pillars, $I/I_{max} \sim 0$. Increasing the field strength to $B = 40$ mT, the pillar structure is significantly enhanced to reach its maximum, $I/I_{max} = 1$; in the top row of the inset, stepwise decreasing B from 40 to 2 mT allows the I/I_{max} to retain with a small drop, $I/I_{max} \sim 0.7$. In contrast to I/I_{max} obtained without training, the trained $I/I_{max} \sim 0$ is significantly facilitated.
(B) Training to suppress the electrical sensitivity of the pillars of the soft ferromagnetic particles by quickly varying (phasic) and slowly varying stimulus (tonic), exploiting the ECFNC assembly/disassembly asymmetry. The shaded area is the error range.

The responses can also be detected using frequency base, using an electrical spike encoder circuit to convert the current output to frequencies, inspired by spike-dependent frequency-based information processing in neuronal systems.[42,43] The sensory currents can therefore be converted to frequencies. A spike encoder, consisting of a ring oscillator, is next designed to encode the electrical sensory current into frequency-encoded electrical spikes (Figure 5).[44,45] Consequently, the perceived field strength in the present range of 2.5–40 mT leads to distinct spiking frequencies (Figures 5A and 5B), realizing the spike rate coding with high fidelity. Beyond this B range, the present magnetic sensation is either not sensitive enough, with no spikes (for $B < 2.5$ mT, as highlighted as a gray background in Figure 5C), or with unresolvably excessive spikes (for $B > 40$ mT, shown as blue background in Figure 5C).

To overcome such sensory frequency range limits in sensing, training the sensitivity of the magnetic sensorics is herein pursued. In biological sensing, spiking adaptations can allow perceiving different intensities of stimuli.[42] In this work, for too-low fields, the sensory training is provided by first training the sensor at a high intermediate B, whereupon promoted sensitivity at low field is achieved (Figures 6A–6C), also revealed as resolvable low-frequency spiking. This allows the recording of

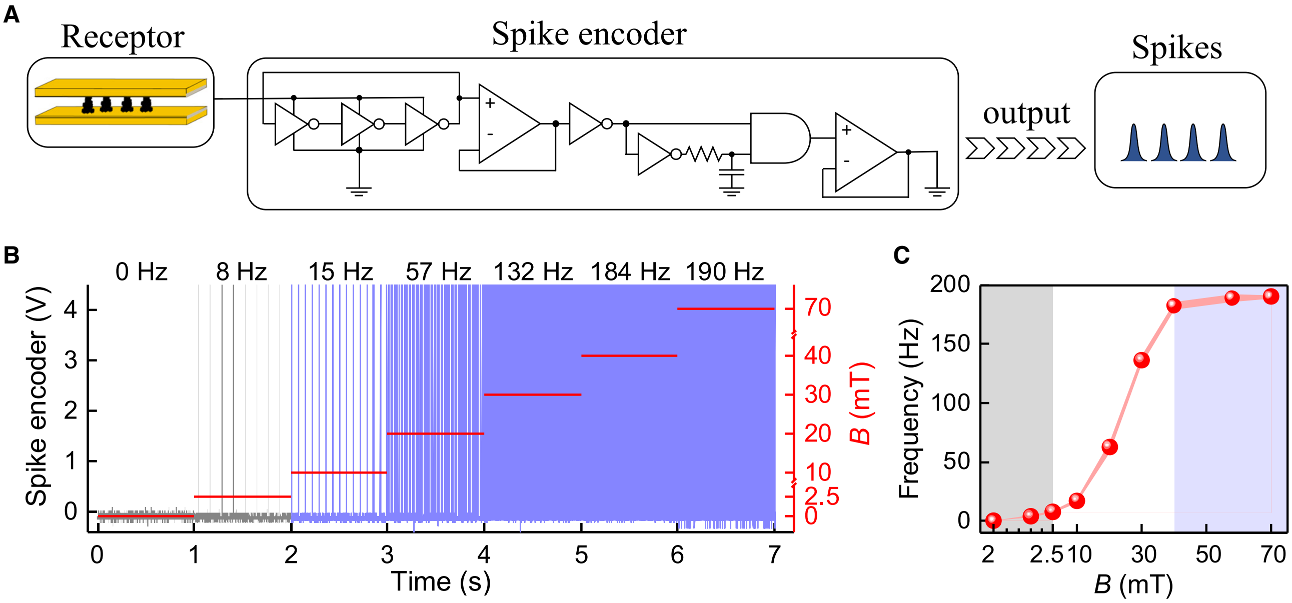

Figure 5. Conversion from the sensoric current domain to frequency domain

(A) Design of systems for artificial magnetic sensation system to allow magnetic field-driven electrical frequency spiking.

(B) Signal transduction from magnetic field to spiking rate. Note that each *B* was applied individually from 0 to target *B* and was separated with a few seconds of *B* = 0 mT to disassemble the pillars. For *B* = 0 mT, no frequency is observed. We omit these information-less data.

(C) A summary of (B). The lower and upper detecting thresholds of the sensor are 2.5 and 40 mT and are highlighted with gray and blue backgrounds, respectively. The shaded area is the error range.

changes at low field conditions. By contrast, at fields higher than 40 mT, training by adaptation to reduced sensitivity is required (Figures 6D–6F), whereupon a lower frequency follows by sensory adaptation by slowly exposing the required field. This permits rescaling and training the dynamic sensory responses in different magnetic field strengths, thus allowing the magnetic field changes to be detected.

The memory allowing trainable sensitivity for the rescaled dynamic range is demonstrated next, using an array of commercial neodymium magnets (instead of electromagnets). A model magnetic landscape is provided by a 3 × 3 matrix that is constituted by nine commercial neodymium magnets with varying geometric and magnetic parameters and embedded into a matrix pattern with magnetic nodes (Figures S13 and S14). The resolved nodes are in the range of 2.5 (dB/dz = 1.8 mT/mm) to 40 mT (dB/dz = 5.7 mT/mm), beyond which they are undiscerned. For example, for a very small value of B = 2 mT (< 2.5 mT), the inherent sensitivity of the present pillars would not be sufficient for detection (Figures 6G and S15). Thanks to the sensory adaptation, the sensitivity threshold can be trained by first imposing the sensor to a strong field B = 40 mT (dB/dz = 5.7 mT/mm) and then approaching slowly toward the node at 2.0 mT field (dB/dz = 1.7 mT/mm), thus allowing spiking frequency to be detected (Figures 6G and S16A). Notably, with this trained new sensitivity, small field value changes near 2.0 mT can be resolved (Figure S16B). By contrast, if the exposed magnetic field is higher than the one possibly resolved by the pristine sensory system (Figure 6H), then training for reduced sensitivity is required. Thus, in this case, the suppressed resolution can be achieved by slowly approaching the nodal point with a high magnetic field (Figures 6H, S16C, and S16D). Consequently, the overall magnetic landscape and changes near the nodes can be mapped (Figure S17). Note that in each cycle of training and sensing, this system requires calibration to gain the actual value of the sensed magnetic field, like

CellPress
OPEN ACCESS

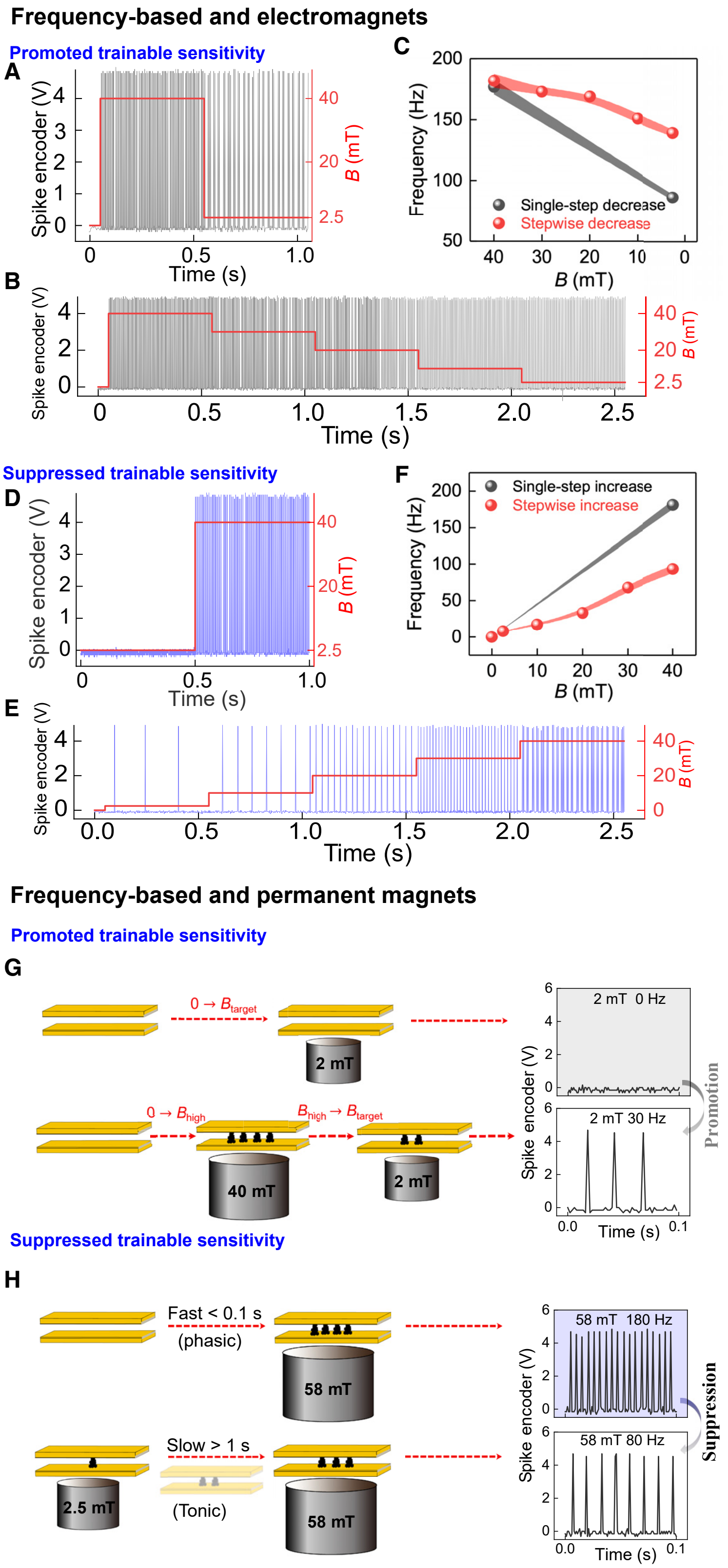

Figure 6. Magnetic sensation spiking systems with trainable sensitivity in the frequency base

(A–C) Sensitivity training inspired by sensory adaptation to promote sensitivity upon using first high intermediate magnetic fields and subsequently decreasing quickly (phasic) or slowly (tonic) to small fields using an electromagnet. (C) The results of multiple tests shown in (A) and (B) are summarized. The shaded areas in (C) are the error ranges.

(D–F) Sensitivity training to suppress sensitivity inspired by sensory adaptation by quickly varying stimulus (phasic) and slowly varying stimulus (tonic), exploiting the particle assembly/disassembly using an electromagnet. (F) The results of multiple tests shown in (D) and (E) are summarized. The shaded areas in (F) are the error ranges.

(G) Sensitivity training to promote sensitivity upon using high intermediate magnetic fields using a permanent magnet toward low magnetic fields (e.g., 2.0 mT [dB/dz = 1.7 mT/mm]).

(H) Sensitivity training to suppress sensitivity inspired by biological quickly varying stimulus (phasic) and slowly varying stimulus (tonic), exploiting the ECFNC assembly/disassembly asymmetry toward high magnetic fields (e.g., at 58 mT [dB/dz = 7.6 mT/mm]).

animate systems in which sensing is always qualitative but adaptable allowing to sense changes. We foresee that trainable magnetic sensing could suggest routes toward bioinspired adjustable sensing of magnetic landscapes, inspired by magnetically navigating animals to detect changes instead of actual values. The approach could allow, for example, conceptually intriguing options for future autonomous navigation of soft robots in imposed magnetic landscapes, as well as introducing magnetoreception function to navigate based on geomagnetic fields concerning the timescale for particle assembling.

In summary, inspired by psychophysical sensory adaptation, we show trainable and adjustable sensory responsiveness toward different ranges of magnetic stimuli via assemblies and disassemblies of pillars consisting of ECFNCs. The hysteresis upon increasing and decreasing B leads to bistable magnetically driven electrical memory. Reference materials with more smooth surfaces show smaller bistability, thus directly underpinning the effect of surface friction and jamming in the reconstruction of the pillars under field changes. Simulations qualitatively explain the behavior. Finally, we demonstrate man-made magnetic sensation protocols, to encode magnetic profiles into bioinspired spiking signals, which show tunable detection limits upon training. This work represents a paradigm in the use of synthetic animate materials to realize lifelike functions and physiological principles and emerges as a promising route stepping into bioinspired complex adaptations.

EXPERIMENTAL PROCEDURES

Resource availability

Lead contact

Further information and requests for resources and reagents should be directed to and will be fulfilled by the lead contact, Bo Peng (pengbo006@gmail.com).

Materials availability

This study did not generate new unique reagents.

Data and code availability

Data and model input script associated with the computational modelling are available at https://doi.org/10.23729/d976204b-2d90-4a5b-9e05-c14b94ae6df3.

Materials synthesis and processing

Ethylene glycol (EG, ≥99.0%) was purchased from Fisher Scientific. Ethanol (≥99.5%) was provided by Altia Oyj. Sodium citrate tribasic dihydrate (NaCit, ≥99.0%), nickel(II) chloride ($NiCl_2$, 98.0%), sodium hydroxide (NaOH, ≥98.0%), and polyethylenimine (PEI, branched, average MW ~25,000) were purchased from

CellPress
OPEN ACCESS

Sigma-Aldrich. Deionized water was used in all of the experiments and obtained from a Millipore Direct-Q UV 3 reverse osmosis filter apparatus. All of the chemicals were used as received.

Preparation of ECFNCs

The synthesis is based on the solvothermal polyol method. Typically, 0.012 mol $NiCl_2$ and 0.006 mol NaCit were dissolved in 30 mL EG by magnetic stirring at 150°C to form a green solution. In parallel, 0.045 mol NaOH was dissolved in 30 mL EG to yield a dark yellow solution. Subsequently, we mixed the $NiCl_2$/NaCit/EG solution with the NaOH/EG in a 100-mL autoclave, heated it at 200°C for 15 h, and then naturally cooled it to room temperature. Finally, the black product was collected by a permanent magnet and washed with ethanol and water repeatedly to remove the retained solvent, then vacuum dried it at room temperature, and stored it in a glove box.

Preparation of soft ferromagnetic Ni colloids with smooth surface

The synthesis is based on the solvothermal polyol method. Typically, 0.004 mol $NiCl_2$ and 0.02 mmol PEI were dissolved in 10 mL EG by magnetic stirring at 150°C to form a deep green solution. In parallel, 0.015 mol NaOH was dissolved in 10 mL EG to yield a dark yellow solution. Subsequently, we mixed the $NiCl_2$/PEI/EG solution with the NaOH/EG in a 50-mL autoclave, heated them at 200°C for 15 h, and then naturally cooled them to room temperature. Finally, the black product was collected by a permanent magnet and washed with ethanol and water repeatedly to remove the retained solvent, then vacuum dried it at room temperature and stored it in a glove box.

Design of device

A pair of Helmholtz coils (GMW 3470) were used to generate a magnetic field opposite to gravity. The generated magnetic fields were detected and calibrated with a Gauss meter (LakeShore 410). The sample powder was sealed and sandwiched between two ITO-coated glass slides. This device was placed in the center between two coils, where the homogeneous magnetic fields can be generated (Figures 2C and S5). The external magnetic field enables the formation of magnetic pillars that bridge the two ITO coatings, allowing for the closure of a circuit. This circuit was connected with an electrometer/high-resistance meter (Keysight B2987A), to which 1 V was applied, and the current (I) was measured simultaneously. The *in situ* observation of the magnetic pillars was carried out with a digital single-lens reflex camera (Nikon D5500).

Artificial magnetic sensation system

The artificial magnetic sensation system comprises two key components, a sensory receptor and a transducer. The assembled and disassembled ECFNCs that were sealed between a pair of ITO-coated glass slides are the sensory receptor, allowing for perceiving the external magnetic fields. A magnetic/electrical circuit inspired by the function of spiking nerve system was designed to encode the sensory information into bioformatted electric pulse signals (Figure 5A). In detail, the ring oscillator converts the direct current signal from the sensory receptor to an alternating current (AC) signal using an odd number of NOT gates. The oscillation frequency of the oscillator output depends on the voltage amplitude input (i.e., the magnetic field applied to the magnetic sensory receptor). The edge detector converts the AC signal to a digital signal (spikes) with identical pulse amplitude and width. The edge detector is made of two NOT gates, a resistor, a capacitor, and an AND gate. The resistor and capacitor are used to delay the input signal and modulate

the output pulse width. The output of the edge detector is connected to an oscilloscope to record the coded information.[44,45]

Trainable sensation of magnetic landscapes

An artificial magnetic landscape was created with a 3 × 3 matrix of neodymium (NdFeB) magnets. The details of each magnet are magnets 1, 8, and 9 (N42 disc magnet, depth [D] 20 mm, height [H] 5 mm), magnet 2 (N45 disc magnet, D 30 mm, H 10 mm), magnet 3 (N42 disc magnet, D 35 mm, H 5 mm), magnets 4 and 6 (N42 cube magnet, length [L] 10 mm), magnet 5 (N52 rod magnet, D 4 mm, H 9 mm), and magnet 7 (N52 rod magnet, D 12 mm, H 12 mm). These nine magnets were placed in a 3 × 3 manner and embedded in a foam board (Figure S13). By moving the sensory receptor above the matrix and then retracting it to allow pillar disassembly, the magnetic field at each pixel of matrix can be sensed and then encoded into bioinspired spiking electric pulses. Thereupon, the magnetic landscape information and local changes therein can be perceived. The parts of the landscape beyond the sensory range (2.5–40 mT) can be perceived via a sensitization/desensitization process. To sense a field smaller than the lower limit, the receptor must be sensitized by first placing it on a strong magnet and then taking it away from the magnet. As such, a small field can be detected. In contrast, the receptor can be desensitized by gradually approaching to the targeted magnet that has a strong magnetic field beyond the upper limit of the sensory receptor (Figures 6G, 6H, and S15–S17).

Characterization

A Zeiss Sigma VP scanning electron microscope operated at 3 kV was used to analyze the surface morphology and obtain a global overview of the ECFNCs. A JEOL JEM-2800 transmission electron microscope with an accelerating voltage of 200 kV was introduced to study the morphology, the size, the EDX, the elemental mapping, and the electron diffraction of the products. Being suitable for SEM and TEM characterization, a droplet of particle aqueous suspension was cast onto silica wafers and carbon-coated copper grids, respectively, allowing the solvent to evaporate at room temperature.

The XRD patterns of the powder samples were recorded with a Rigaku SmartLab X-ray diffraction using Cu Kα radiation (λ = 0.15406 nm) at 35 kV and 15 mA to analyze the crystal structure at a scanning rate of 0.5° min^{-1} in a 2θ angle from 30° to 90°.

The magnetic properties were measured with a MPMS-XL7 device by Quantum Design, which integrates a superconducting quantum interference device (SQUID) magnetometer. The magnetic movement and susceptibility were measured at 300 and 360 K, respectively, with an applied field between −800 and 800 mT. For ZFC and FC measurements, the samples were initially cooled in a zero field to 5 K. With an applied field of 100 Oe, the susceptibility was recorded as a ZFC curved by increasing the temperature. After the temperature had reached 350 K, the samples were progressively cooled, and the susceptibility was recorded as an FC curve.

Simulations

We consider the ECFNCs to form aggregates, granular assemblies with grains of different preferential magnetization directions. Such aggregate particles can be assumed to follow Newton's equations of motion, namely,

$$\sum_j \mathbf{F}_j = m a(\mathbf{r}, t). \qquad \text{(Equation 1)}$$

Here, $\sum_j \mathbf{F}_j$ is the sum of all forces acting on the particle, m its mass, and $\mathbf{a}(\mathbf{r},t)$ its acceleration at position $\mathbf{r}$ and time t. The effect of the surrounding medium can be mimicked by introducing a stochastic random force together with a frictional term, leading to

$$m\mathbf{a}(\mathbf{r},t) = m\frac{d\mathbf{v}(\mathbf{r},t)}{dt} = -\gamma\mathbf{v}(\mathbf{r},t) + \boldsymbol{\xi}(t). \quad \text{(Equation 2)}$$

Here, γ is the friction coefficient, $\mathbf{v}$ the velocity of the particle, and $\boldsymbol{\xi}$ a random noise term that satisfies $\langle\xi(t)\rangle = 0$ and $\langle\xi(t)\xi(t')^T\rangle = 2\gamma k_B m T\delta(t-t')$, where T is the temperature and k_B the Boltzmann constant, following the fluctuation/dissipation theorem.[46] For $\mathcal{N}$ interacting particles, with the potential $\Phi_i(\mathbf{r}_i)$ capturing both interparticle interactions and external forces, Equation 2 leads to coupled equations of motion corresponding to

$$m_i\frac{d\mathbf{v}_i(\mathbf{r}_i,t)}{dt} = -\gamma_i\mathbf{v}_i(\mathbf{r}_i,t) + \boldsymbol{\xi}_i(t) - \nabla\Phi_i(\mathbf{r}_i). \quad \text{(Equation 3)}$$

For identical particles, Equation 3 simplifies to

$$m\frac{d\mathbf{v}_i(\mathbf{r}_i,t)}{dt} = -\gamma\mathbf{v}_i(\mathbf{r}_i,t) + \boldsymbol{\xi}(t) - \nabla\Phi_i(\mathbf{r}_i), \quad \text{(Equation 4)}$$

known as the Langevin equation of motion.[47] In the overdamped regime, it reduces to

$$\gamma\mathbf{v}_i(\mathbf{r}_i,t) = \boldsymbol{\xi}(t) - \nabla\Phi_i(\mathbf{r}_i). \quad \text{(Equation 5)}$$

In this, the velocity relaxation time has been assumed to be more rapid than the position relaxation time. Equation 5 can be solved numerically. Here, the Euler-Maruyama integration algorithm

$$\mathbf{r}_i(t+dt) = \mathbf{r}_i(t) - \gamma^{-1}\nabla\Phi_i(\mathbf{r}_i)dt + \delta\mathbf{r} \quad \text{(Equation 6)}$$

was used. In this, $\delta\mathbf{r}$ is the random displacement term with standard deviation $\sqrt{2k_B T\gamma^{-1/2}}$. The time evolution resulting from the discrete numerical integration of the equations of motion is commonly referred to as Brownian dynamics simulations (algorithm).

The potential $\Phi_i(\mathbf{r}_i)$ models all interparticle interactions and external forces contributions acting on the particles. In this system, the most important contributions to the potential for capturing the particle assembly response are gravity, their interactions with the ITO surface and the external magnetic field B, as well as intraparticle interactions resulting from the dipole-dipole and steric interactions. In addition, residual deviations from a perfect dipole due to granular character of the assembly particle should be considered. Gravity is modeled via the potential

$$\Phi_i^g(z_i) = mgz_i, \quad \text{(Equation 7)}$$

where g is the gravitational constant. To provide a sensible model for the interaction with the ITO surfaces used in the experiments, a Lennard-Jones (LJ) 10-4-3 potential

$$\Phi_i^{LJ}(z_i) = 2\pi\tilde{\epsilon}\left[\frac{2}{5}\cdot\frac{\tilde{\sigma}^{10}}{z_i^{10}} - \frac{\tilde{\sigma}^4}{z_i^4} - \frac{\sqrt{2}\tilde{\sigma}^3}{3\left(z_i+\left(\frac{0.61\tilde{\sigma}}{\sqrt{2}}\right)\right)^3}\right] \quad \text{(Equation 8)}$$

is used. Here $\tilde{\sigma}$ and $\tilde{\epsilon}$ are the standard LJ parameters.[48] We set $\tilde{\sigma} = 2\sigma$, where $\sigma = 1$ is the diameter of the particles, also setting the length scale of the system. The energy scale is set by $\epsilon = 1$ and $\tilde{\epsilon} = 0.5\epsilon$. Finally, the coupling between magnetic dipole moments $\mathbf{m}_i$ of the particles and external magnetic field B is modeled by

$$\Phi_i^{\mathrm{B}}(\mathbf{r}_i) = -\mathbf{m}_i \cdot \mathbf{B}(\mathbf{r}_i). \quad \text{(Equation 9)}$$

The magnetic dipole moment of particle *i* interacting with the one of particle *j* contributes to the potential energy by

$$\Phi_{ij}^{\mathrm{dd}}(\mathbf{r}_i, \mathbf{r}_j) = \frac{1}{r^3}(\mathbf{m}_i \cdot \mathbf{m}_j)(\mathbf{r}_i - \mathbf{r}_j) - \frac{3}{r^5}(\mathbf{m}_i \cdot (\mathbf{r}_i - \mathbf{r}_j))(\mathbf{m}_j \cdot (\mathbf{r}_i - \mathbf{r}_j)) \quad \text{(Equation 10)}$$

In this, $r = \|\mathbf{r}_i - \mathbf{r}_j\|$. We assume the modeled particles to represent granular aggregates of ECFNCs. Magnetic characteristics of such granular particles are not captured by a single, unique dipole moment. Instead, each grain contributes a dipole moment, leading to a collection of dipole moments varying in strength and direction. This results in a preferential magnetization direction and a net dominant dipole moment, modeled above by $\mathbf{m}$. The individual components, however, contribute deviation, which we model as an effectively isotropic, attractive interaction described by

$$\Phi_{ij}^{\mathrm{Yuk}}(r) = \frac{A}{\kappa} e^{-\kappa(r-\sigma)}. \quad \text{(Equation 11)}$$

In this, κ controls the decay of the interaction, and A is the strength.

The orientation of the dipole moment in the simulation time trajectory follows the equation

$$\mathbf{m}_i(t + dt) = \frac{\mathbf{m}_i(t) + \omega_i \times \mathbf{m}_i(t)dt}{|\mathbf{m}_i(t) + \omega_i \times \mathbf{m}_i(t)|}, \quad \text{(Equation 12)}$$

where the dynamics are captured by ω_i defined as

$$\omega_i = \frac{\mathbf{T}_i}{\gamma_\mathrm{r}} + \sqrt{\frac{2k_\mathrm{B}T_\mathrm{r}}{\gamma_\mathrm{r}}\frac{d\mathbf{W}}{dt}}. \quad \text{(Equation 13)}$$

Here, $\mathbf{T}_i$ is the torque affecting the dipole $\mathbf{m}_i$, γ_r is the rotational friction coefficient, T_r is the rotational temperature, and $d\mathbf{W}$ is a random vector generated to emulate Brownian motion. The nonstochastic part of Equation 13 depends on the torque induced by the dipole-dipole interaction

$$\mathbf{T}_{ij}^{\mathrm{dd}} = -\frac{1}{r^3}(\mathbf{m}_i \times \mathbf{m}_j) + \frac{3}{r^5}(\mathbf{m}_j \cdot (\mathbf{r}_i - \mathbf{r}_j))(\mathbf{m}_i \times (\mathbf{r}_i - \mathbf{r}_j)), \quad \text{(Equation 14)}$$

as well as the torque induced by the external field

$$\mathbf{T}_i^{\mathrm{B}} = \mathbf{m}_i \times \mathbf{B}(\mathbf{r}_i). \quad \text{(Equation 15)}$$

The simulations were run using LAMMPS (Large-Scale Atomic/Molecular Massively Parallel Simulator).[49] The system under consideration consists of $\mathcal{N} = 1,440$ colloidal particles with a dipole moment of $\left|\left|\mathbf{m}\right|\right|(4\pi\varepsilon_0\sigma^3\epsilon)^{-1/2} = 1$ in a $(48\sigma)^3$ simulations box. Periodic boundary conditions along the *xy* plane were used. To model the standard air conditions around the particles, we set $k_\mathrm{B}T\epsilon^{-1} = 0.1$ and $k_\mathrm{B}T_\mathrm{r}\epsilon^{-1} = 0.01$, whereas the mass of the particles is set to $m = 1$. These choices are made to decrease the thermal fluctuations of the Brownian motion, as one may expect in a dilute surrounding medium such as air. The variables of Equation 13 are set to $A\sigma\epsilon^{-1} = -0.2$ and $\kappa\sigma = 1$, respectively. The translational friction coefficient is fixed at $\gamma t m^{-1} = 10$, whereas the rotational friction coefficient γ_r is varied. The integration time step is set to $\mathrm{dt} = 10^{-4}\tau^*$, where τ^* is defined as $\tau^* = t\sqrt{\epsilon m^{-1}\sigma^{-2}}$. The particles are initialized to random positions in the simulations box, with the dipole moment turned off. Setting $g\,\frac{m\sigma}{\epsilon} = 10$ for 10^7 time steps allowed the particles to settle on the ITO surface at the bottom of the box. After that, the value of g was decreased to $g\,\frac{m\sigma}{\epsilon} = 0.05$ and additional 10^7 time steps simulated to achieve

equilibration (monitored by local density equilibration). The dipole moments were then turned on, set in random orientation.

Three different simulations were run with differing values of rotational friction coefficient γ_r, namely $\gamma^* = \gamma_r t m^{-1} \sigma^{-2} = 30, 1{,}000$ and $3{,}000$, respectively. For Figure 3B, simulations were run for 27×10^7 time steps with the magnetic field strength increasing to $B^* = B\sqrt{4\pi\varepsilon_0\sigma^3\epsilon^{-1}} = 12$ in $\Delta B^* = 2$ increments every 3×10^7 time steps starting from $B^* = 0$. At maximum value of $B^* = 12$, additional 3×10^7 time steps were simulated, after which, the field was turned off for the rest of the simulation.

For Figure 3C, the simulations were run for 21×10^7 time steps, with the magnetic field strength increasing to $B^* = 10$ starting from $B^* = 0$. The field was then reversed first to a value of $B^* = -10$ and then back to a value of $B^* = 10$, all in increments of $\Delta B^* = 2.5$ lasting 10^7 time steps each.

Note that the stable version of LAMMPS used here (June 23, 2022 stable release) does not currently provide the implementation of a magnetic field. To bypass this practical issue, we use the external electric field function, which results in the same effect (torque) on the dipole moments of the particles. Note that this solution is possible only if the modeled system does not consider any point charges.

SUPPLEMENTAL INFORMATION

Supplemental information can be found online at https://doi.org/10.1016/j.xcrp.2024.101923.

ACKNOWLEDGMENTS

The authors acknowledge the facilities and technical support provided by Aalto University OtaNano - Nanomicroscopy Center and the assistance from Jiahui Kang for TEM imaging. This work was supported by ERC (Advanced Grant DRIVEN, no. 742829), the Academy of Finland (nos. 321443, 328942, and 316973, and Center of Excellence Program of Life-Inspired Hybrid Materials, projects nos. 346108 and 346111), the China Scholarship Council (no. 201906310146), and the Swiss National Science Foundation (no. P500PT_206916) (to A.S.). Computational resources by CSC IT Center for Finland, Aalto Science-IT and RAMI – RawMatters Finland Infrastructure are also gratefully acknowledged.

AUTHOR CONTRIBUTIONS

B.P. and O.I. initiated the project. B.P., H.T., and O.I. conceived the idea and designed the experiments. X.L. performed the experiments and analyzed the data with the assistance of H.T., X.H., and M.C. E.S. conducted the simulations and their analysis, instructed by A.S., who developed the simulations model—both under the supervision of M.S. S.v.D. supervised H.T. B.P. and O.I. supervised the project. X.L., B.P., and O.I. wrote the paper, with contributions from all of the coauthors.

DECLARATION OF INTERESTS

The authors declare no competing interests.

Received: September 27, 2023
Revised: February 7, 2024
Accepted: March 18, 2024
Published: April 4, 2024

REFERENCES

1. Fratzl, P., Jacobs, K., Möller, M., Scheibel, T., and Sternberg, K. (2020). Materials research: inspired by nature: innovation potential of biologically inspired materials. Acatech-National Academy of Science and Engineering. https://en.acatech.de/publication/materials-research/.

2. Wegst, U.G.K., Bai, H., Saiz, E., Tomsia, A.P., and Ritchie, R.O. (2015). Bioinspired structural materials. Nat. Mater. *14*, 23–36. https://doi.org/10.1038/nmat4089.

3. Bhushan, B. (2009). Biomimetics: lessons from nature - an overview. Philos. Trans. R. Soc. A *367*, 1445–1486. https://doi.org/10.1098/rsta.2009.0011.

4. Studart, A.R. (2012). Towards High-Performance Bioinspired Composites. Adv. Mater. *24*, 5024–5044. https://doi.org/10.1002/adma.201201471.

5. Morits, M., Verho, T., Sorvari, J., Liljeström, V., Kostiainen, M.A., Gröschel, A.H., and Ikkala, O. (2017). Toughness and Fracture Properties in Nacre-Mimetic Clay/Polymer Nanocomposites. Adv. Funct. Mater. *27*, 1605378. https://doi.org/10.1002/adfm.201605378.

6. Wang, D., Sun, Q., Hokkanen, M.J., Zhang, C., Lin, F.Y., Liu, Q., Zhu, S.P., Zhou, T., Chang, Q., He, B., et al. (2020). Design of robust superhydrophobic surfaces. Nature *582*, 55–59. https://doi.org/10.1038/s41586-020-2331-8.

7. Droguet, B.E., Liang, H.L., Frka-Petesic, B., Parker, R.M., De Volder, M.F.L., Baumberg, J.J., and Vignolini, S. (2022). Large-scale fabrication of structurally coloured cellulose nanocrystal films and effect pigments. Nat. Mater. *21*, 352–358. https://doi.org/10.1038/s41563-021-01135-8.

8. Nepal, D., Kang, S., Adstedt, K.M., Kanhaiya, K., Bockstaller, M.R., Brinson, L.C., Buehler, M.J., Coveney, P.V., Dayal, K., El-Awady, J.A., et al. (2023). Hierarchically structured bioinspired nanocomposites. Nat. Mater. *22*, 18–35. https://doi.org/10.1038/s41563-022-01384-1.

9. Kontturi, E., Laaksonen, P., Linder, M.B., Ikkala, O., Nonappa, Groschel, A.H., Gröschel, A.H., and Rojas, O.J. (2018). Advanced Materials through Assembly of Nanocelluloses. Adv. Mater. *30*, 1703779. https://doi.org/10.1002/adma.201703779.

10. Harrington, M.J., and Fratzl, P. (2021). Natural load-bearing protein materials. Prog. Mater. Sci. *120*, 100767. https://doi.org/10.1016/j.pmatsci.2020.100767.

11. Ball, P. (2021). Coming alive. Nat. Mater. *20*, 285. https://doi.org/10.1038/s41563-021-00949-w.

12. Kaspar, C., Ravoo, B.J., van der Wiel, W.G., Wegner, S.V., and Pernice, W.H.P. (2021). The rise of intelligent matter. Nature *594*, 345–355. https://doi.org/10.1038/s41586-021-03453-y.

13. Sitti, M. (2021). Physical intelligence as a new paradigm. Extreme Mech. Lett. *46*, 101340. https://doi.org/10.1016/j.eml.2021.101340.

14. Kandel, E.R. (2007). In Search of Memory: The Emergence of a New Science of Mind (WW Norton & Company).

15. McBurney, D.H., and Balaban, C.D. (2009). A heuristic model of sensory adaptation. Atten. Percept. Psychophys. *71*, 1941–1961. https://doi.org/10.3758/APP.71.8.1941.

16. Adibi, M., and Lampl, I. (2021). Sensory Adaptation in the Whisker-Mediated Tactile System: Physiology, Theory, and Function. Front. Neurosci. *15*, 770011. https://doi.org/10.3389/fnins.2021.770011.

17. Walther, A. (2020). Viewpoint: From Responsive to Adaptive and Interactive Materials and Materials Systems: A Roadmap. Adv. Mater. *32*, 1905111. https://doi.org/10.1002/adma.201905111.

18. Zhang, H., Zeng, H., Priimagi, A., and Ikkala, O. (2020). Viewpoint: Pavlovian Materials-Functional Biomimetics Inspired by Classical Conditioning. Adv. Mater. *32*, 1906619. https://doi.org/10.1002/adma.201906619.

19. Sato, Y., and Aihara, K. (2011). A Bayesian Model of Sensory Adaptation. PLoS One *6*, e19377. https://doi.org/10.1371/journal.pone.0019377.

20. Zhang, H., Zeng, H., Priimagi, A., and Ikkala, O. (2019). Programmable responsive hydrogels inspired by classical conditioning algorithm. Nat. Commun. *10*, 3267. https://doi.org/10.1038/s41467-019-11260-3.

21. Zeng, H., Zhang, H., Ikkala, O., and Priimagi, A. (2020). Associative Learning by Classical Conditioning in Liquid Crystal Network Actuators. Matter *2*, 194–206. https://doi.org/10.1016/j.matt.2019.10.019.

22. Liu, X., Tan, H., Rigoni, C., Hartikainen, T., Asghar, N., van Dijken, S., Timonen, J.V.I., Peng, B., and Ikkala, O. (2022). Magnetic field-driven particle assembly and jamming for bistable memory and response plasticity. Sci. Adv. *8*, eadc9394. https://doi.org/10.1126/sciadv.adc9394.

23. Sangwan, V.K., and Hersam, M.C. (2020). Neuromorphic nanoelectronic materials. Nat. Nanotechnol. *15*, 517–528. https://doi.org/10.1038/s41565-020-0647-z.

24. Stuart, M.A.C., Huck, W.T.S., Genzer, J., Müller, M., Ober, C., Stamm, M., Sukhorukov, G.B., Szleifer, I., Tsukruk, V.V., Urban, M., et al. (2010). Emerging applications of stimuli-responsive polymer materials. Nat. Mater. *9*, 101–113. https://doi.org/10.1038/nmat2614.

25. Herbert, K.M., Schrettl, S., Rowan, S.J., and Weder, C. (2017). 50th Anniversary Perspective: Solid-State Multistimuli, Multiresponsive Polymeric Materials. Macromolecules *50*, 8845–8870. https://doi.org/10.1021/acs.macromol.7b01607.

26. Wei, M., Gao, Y., Li, X., and Serpe, M.J. (2017). Stimuli-responsive polymers and their applications. Polym. Chem. *8*, 127–143. https://doi.org/10.1039/C6PY01585A.

27. Lendlein, A., and Gould, O.E.C. (2019). Reprogrammable recovery and actuation behaviour of shape-memory polymers. Nat. Rev. Mater. *4*, 116–133. https://doi.org/10.1038/s41578-018-0078-8.

28. Xia, X., Spadaccini, C.M., and Greer, J.R. (2022). Responsive materials architected in space and time. Nat. Rev. Mater. *7*, 683–701. https://doi.org/10.1038/s41578-022-00450-z.

29. Schattling, P., Jochum, F.D., and Theato, P. (2014). Multi-stimuli responsive polymers–the all-in-one talents. Polym. Chem. *5*, 25–36. https://doi.org/10.1039/C3PY00880K.

30. Hu, W., Lum, G.Z., Mastrangeli, M., and Sitti, M. (2018). Small-scale soft-bodied robot with multimodal locomotion. Nature *554*, 81–85. https://doi.org/10.1038/nature25443.

31. Johnsen, S., and Lohmann, K.J. (2008). Magnetoreception in animals. Phys. Today *61*, 29–35. https://doi.org/10.1063/1.2897947.

32. Mora, C.V., and Bingman, V.P. (2013). Detection of Magnetic Field Intensity Gradient by Homing Pigeons (Columba livia) in a Novel "Virtual Magnetic Map'' Conditioning Paradigm. PLoS One *8*, e72869. https://doi.org/10.1371/journal.pone.0072869.

33. Peng, B., Zhang, X., Aarts, D.G.A.L., and Dullens, R.P.A. (2018). Superparamagnetic nickel colloidal nanocrystal clusters with antibacterial activity and bacteria binding ability. Nat. Nanotechnol. *13*, 478–482. https://doi.org/10.1038/s41565-018-0108-0.

34. Frenkel, J., and Doefman, J. (1930). Spontaneous and induced magnetisation in ferromagnetic bodies. Nature *126*, 274–275. https://doi.org/10.1038/126274a0.

35. Zhang, H., and Widom, M. (1995). Field-induced forces in colloidal particle chains. Phys. Rev. E *51*, 2099–2103. https://doi.org/10.1103/PhysRevE.51.2099.

36. Ikeda, H., Brito, C., Wyart, M., and Zamponi, F. (2020). Jamming with tunable roughness. Phys. Rev. Lett. *124*, 208001. https://doi.org/10.1103/PhysRevLett.124.208001.

37. Derjaguin, B.V., Muller, V.M., and Toporov, Y. (1975). Effect of contact deformations on the adhesion of particles. J. Colloid Interface Sci. *53*, 314–326. https://doi.org/10.1016/0021-9797(75)90018-1.

38. Delong, S., Balboa Usabiaga, F., and Donev, A. (2015). Brownian dynamics of confined rigid bodies. J. Chem. Phys. *143*, 144107. https://doi.org/10.1063/1.4932062.

39. Somervail, R., Zhang, F., Novembre, G., Bufacchi, R.J., Guo, Y., Crepaldi, M., Hu, L., and Iannetti, G.D. (2021). Waves of change: brain sensitivity to differential, not absolute, stimulus intensity is conserved across humans and rats. Cerebr. Cortex *31*, 949–960. https://doi.org/10.1093/cercor/bhaa267.

40. Ritz, T., Adem, S., and Schulten, K. (2000). A model for photoreceptor-based magnetoreception in birds. Biophys. J. *78*,

707–718. https://doi.org/10.1016/S0006-3495(00)76629-X.

41. Sedgwick, J., Michalson, W.R., and Ludwig, R. (1998). Design of a digital gauss meter for precision magnetic field measurements. IEEE Trans. Instrum. Meas. *47*, 972–977. https://doi.org/10.1109/19.744652.

42. Nikola, K. (2018). Springer Series on Bio- and Neurosystems (Springer).

43. Jung, Y.H., Park, B., Kim, J.U., and Kim, T.I. (2019). Bioinspired electronics for artificial sensory systems. Adv. Mater. *31*, 1803637. https://doi.org/10.1002/adma.201803637.

44. Tan, H., Tao, Q., Pande, I., Majumdar, S., Liu, F., Zhou, Y., Persson, P.O.Å., Rosen, J., and van Dijken, S. (2020). Tactile sensory coding and learning with bio-inspired optoelectronic spiking afferent nerves. Nat. Commun. *11*, 1–9. https://doi.org/10.1038/s41467-019-13827-6.

45. Tan, H., Zhou, Y., Tao, Q., Rosen, J., and van Dijken, S. (2021). Bioinspired multisensory neural network with crossmodal integration and recognition. Nat. Commun. *12*, 1120–1129. https://doi.org/10.1038/s41467-021-21404-z.

46. Kubo, R. (1966). The fluctuation-dissipation theorem. Rep. Prog. Phys. *29*, 255–284. https://doi.org/10.1088/0034-4885/29/1/306.

47. Langevin, P. (1908). Sur la théorie du mouvement brownien (On the Theory of Brownian Motion). C. R. Acad. Sci. *146*, 530–533.

48. Magda, J.J., Tirrell, M., and Davis, H.T. (1985). Molecular dynamics of narrow, liquid-filled pores. J. Chem. Phys. *83*, 1888–1901. https://doi.org/10.1063/1.449375.

49. Thompson, A.P., Aktulga, H.M., Berger, R., Bolintineanu, D.S., Brown, W.M., Crozier, P.S., J.in't Veld, P., in 't Veld, P.J., Kohlmeyer, A., Moore, S.G., et al. (2022). LAMMPS-a flexible simulation tool for particle-based materials modeling at the atomic, meso, and continuum scales. Comput. Phys. Commun. *271*, 108171. https://doi.org/10.1016/j.cpc.2021.108171.

Cell Reports Physical Science, Volume *5*

Supplemental information

Trainable bioinspired magnetic sensitivity adaptation using ferromagnetic colloidal assemblies

Xianhu Liu, Hongwei Tan, Emil Stråka, Xichen Hu, Min Chen, Sebastiaan van Dijken, Alberto Scacchi, Maria Sammalkorpi, Olli Ikkala, and Bo Peng

Supplemental Information

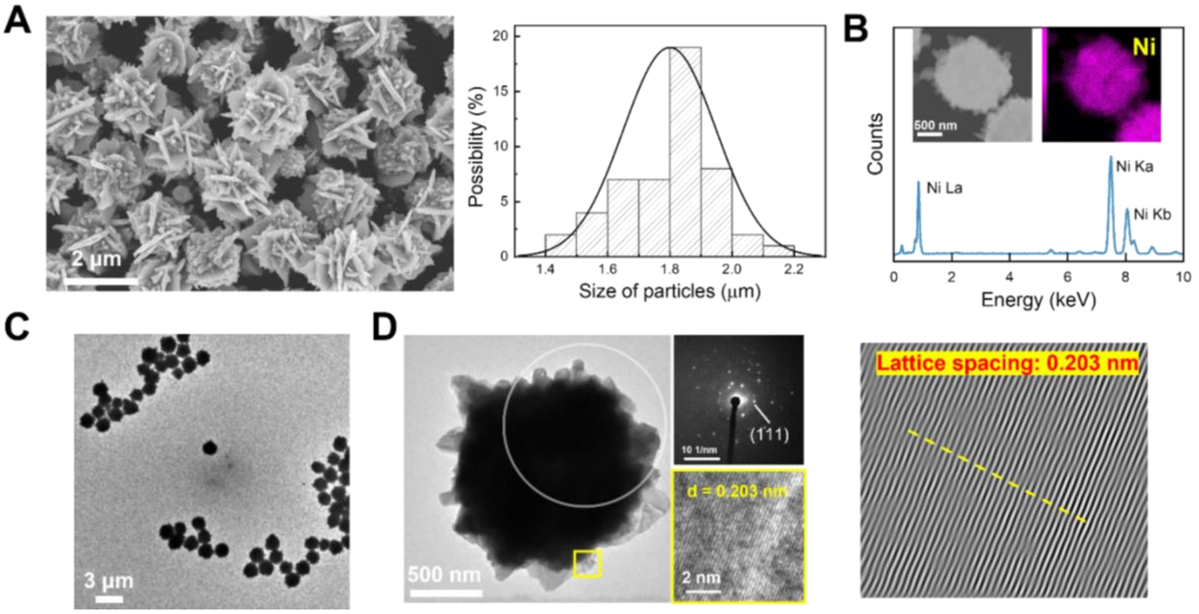

Figure S1. Characterization of ECFNCSs.

A, SEM, **B**, EDX, **C**, TEM and **D**, HRTEM of ECFNCs. SEM image shows the flower-like rough surface of ECFNCs and the quantitative statistics reveals the average diameters of ~1.8 μm. Energy-dispersive X-ray spectroscopy and elemental mapping confirm the nickel nature of the particles. Analysis of HRTEM result confirms that the ECFNCs are comprised of *fcc* nickel nanocrystals.

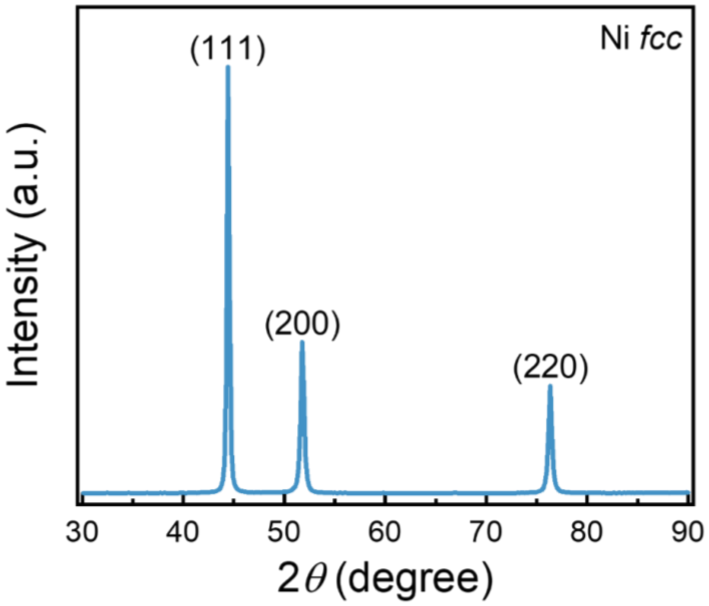

Figure S2. XRD characterization of ECFNCs.

X-ray diffraction unravels the *fcc* crystalline structure of ECFNCs.

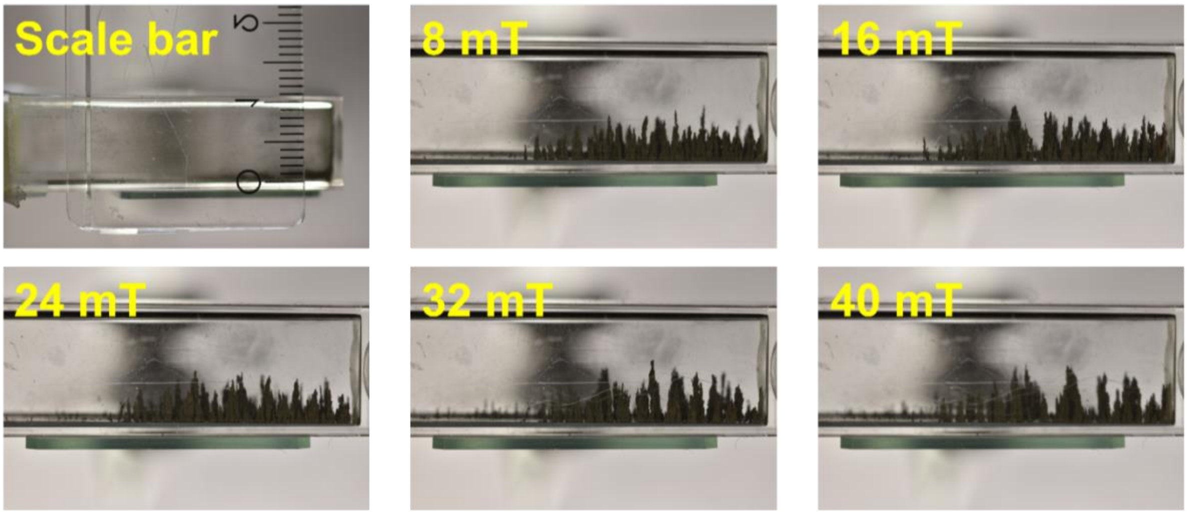

Figure S3. The unconstrained assembly of ECFNCs.

The side view images of pillars upon application of magnetic field *B*.

Table S1. The summarized details of unconstrained pillars at different magnetic fields.

B (mT)	Data 1		Data 2		Data 3		Data 4		Data 5		**Average**	
	No. of pillars	*H* (mm)	No. of pillars	*H* (mm)	No. of pillars	*H* (mm)	No. of pillars	*H* (mm)	No. of pillars	*H* (mm)	No. of pillars	*H* (mm)
8	36	2.765	29	2.823	29	2.791	31	2.647	27	2.706	30.4 **30**	**2.7464**
16	35	2.956	29	3.019	27	2.968	27	2.682	21	2.716	27.8 **28**	**2.8682**
24	32	3.030	24	3.246	26	3.051	26	3.028	20	2.958	25.6 **26**	**3.0626**
32	28	3.361	22	3.456	23	3.106	24	3.271	19	3.106	23.2 **23**	**3.2600**
40	23	3.537	23	3.629	20	3.464	24	3.347	18	3.630	21.6 **22**	**3.5214**

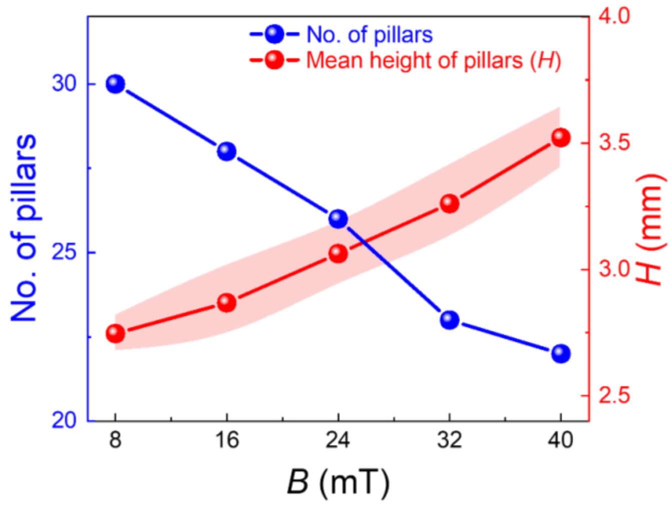

Figure S4. The height and population of unconstrained pillars as a function of *B*.

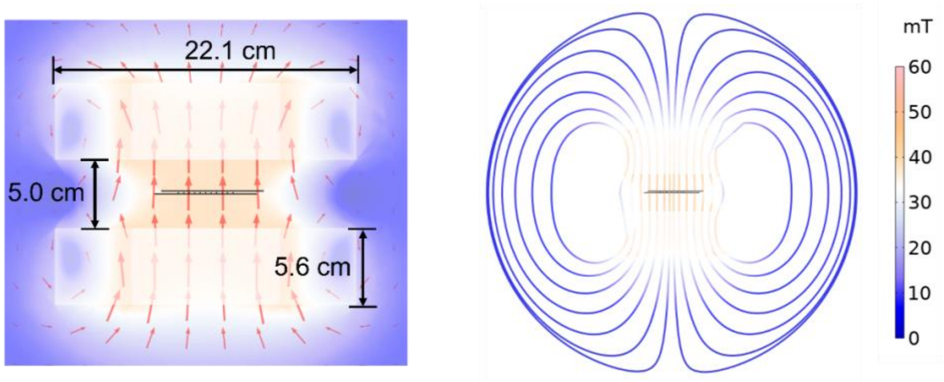

Figure S5. Simulated magnetic field profile.

The simulated magnetic fields generated by a pair of Helmholtz coils, and the magnified field profile around the sample site.

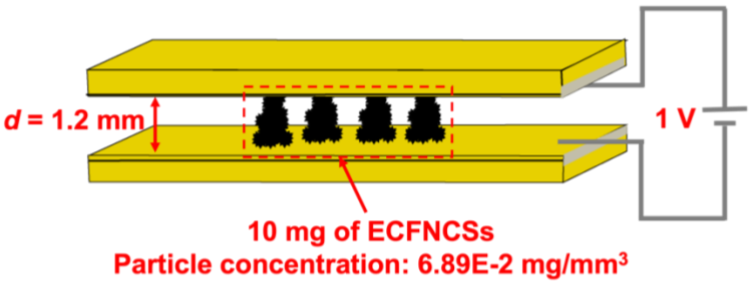

Figure S6. Schematic of the electric cell used in the experiment.

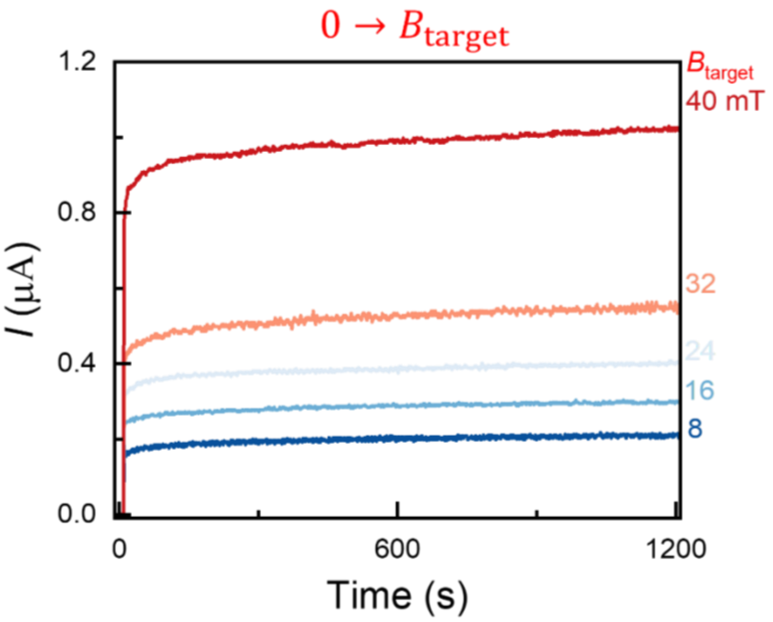

Figure S7. Electric performance during the process of 0 → $B_{\mathbf{target}}$.

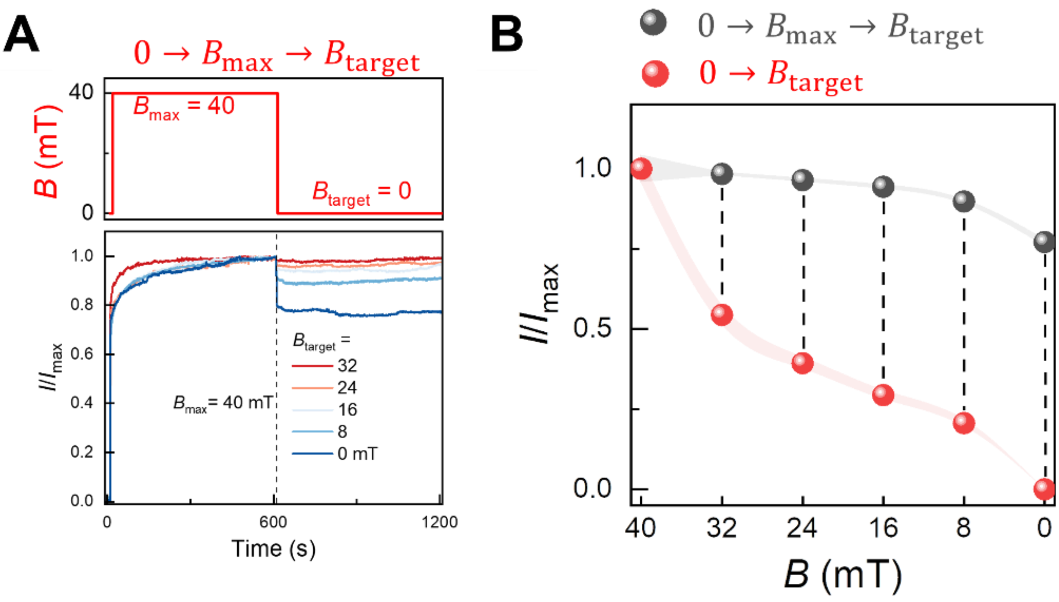

Figure S8. Suppressed current based memory.

A, Electric performance during the process of 0 → B_{max} → B_{target}. **B,** The disassembly of magnetic pillars by first applying B_{max} at 40 mT for 600 s and then decreasing it to B_{target} (0 → B_{max} → B_{target}, grey dots), in comparison to those (red dots) acquired using 0 → B_{target}.

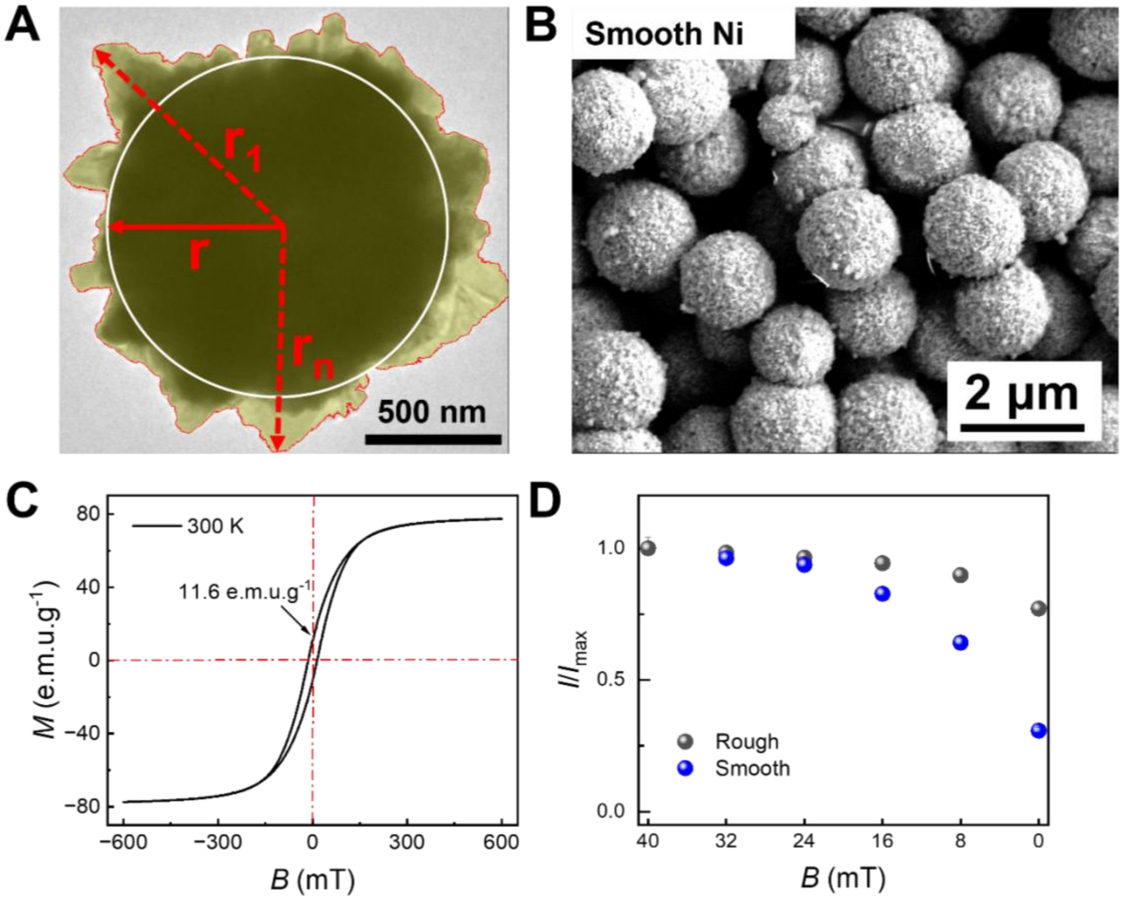

Figure S9. Characterizations of nickel particles.

A, The quantification of the particle's surface roughness. It simply defined by calculating the average value of r/r_n. **B,** SEM image of soft ferromagnetic Ni particles with smooth surface, their mean diameter is 1.6 μm. **C,** Magnetic moment *M* of smooth Ni particles as a function of *B*. **D,** The disassembly of rough particles ECFNCs and smooth Ni particles by first applying B_{max} at 40 mT for 600 s and then decreasing it to different B_{target}.

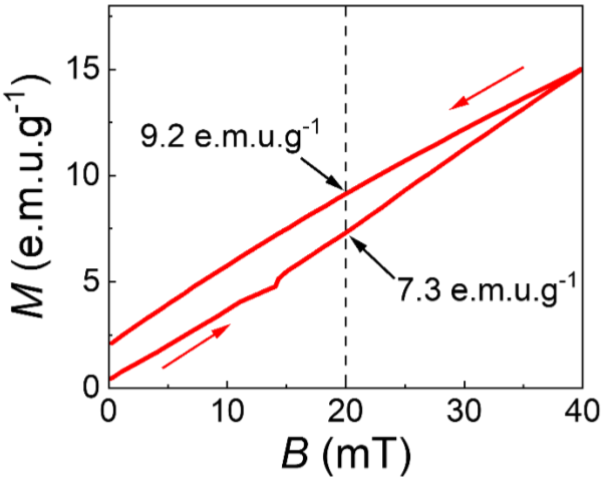

Figure S10. Mass magnetic moment *M* as a function of external magnetic *B*.

The *M* value measured after 0 → 40 mT → 20 mT process (9.2 e.m.u.g^{-1}) is higher than that of directly increasing *B* to 20 mT (7.3 e.m.u.g^{-1}).

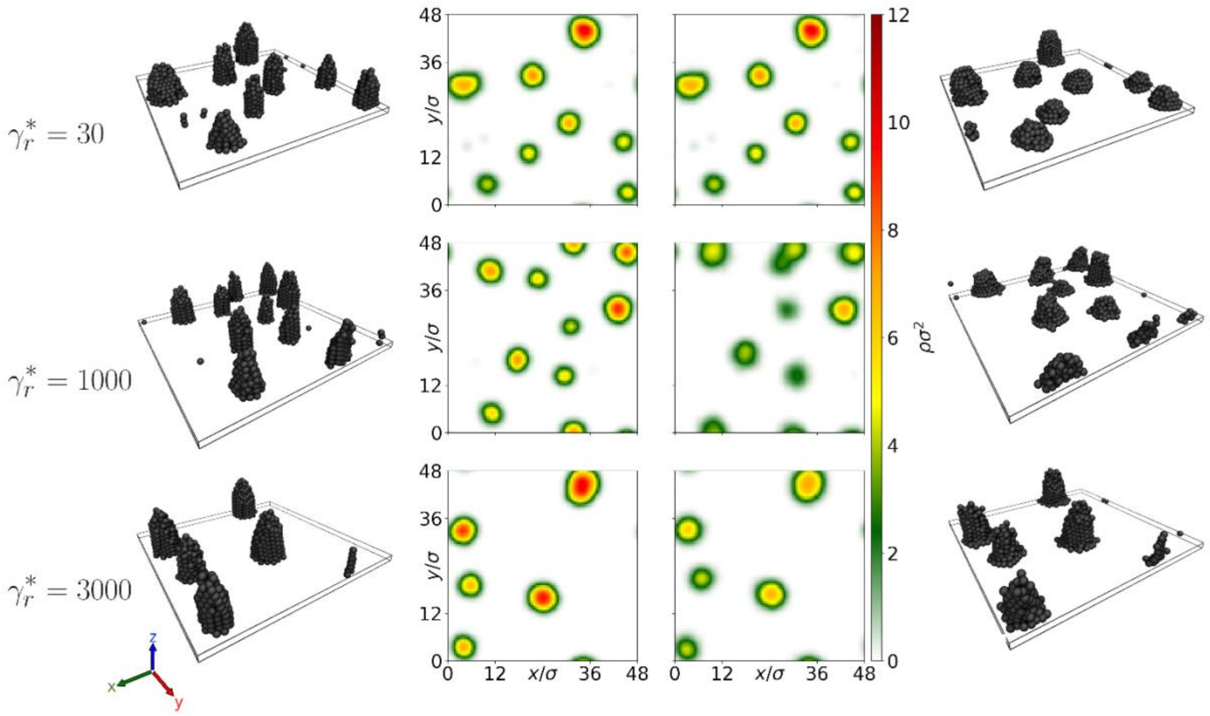

Figure S11. The effect of rotational friction γ_r^* on structural memory in the simulations. At left, visualizations and density profiles corresponding to main manuscript Fig. 3B at reduced simulation time 21 (at peak magnetization) and at right, the corresponding data at time 27 (after magnetic field retraction). Reduced simulation time is measured in units of 10^7 time steps. Magnetic field strength corresponds to Fig. 3B. Increasing the rotational friction between particles enhances the structural retention after the magnetic field is retracted. $\rho\sigma^2$ is two-dimensional dimensionless number density of particles.

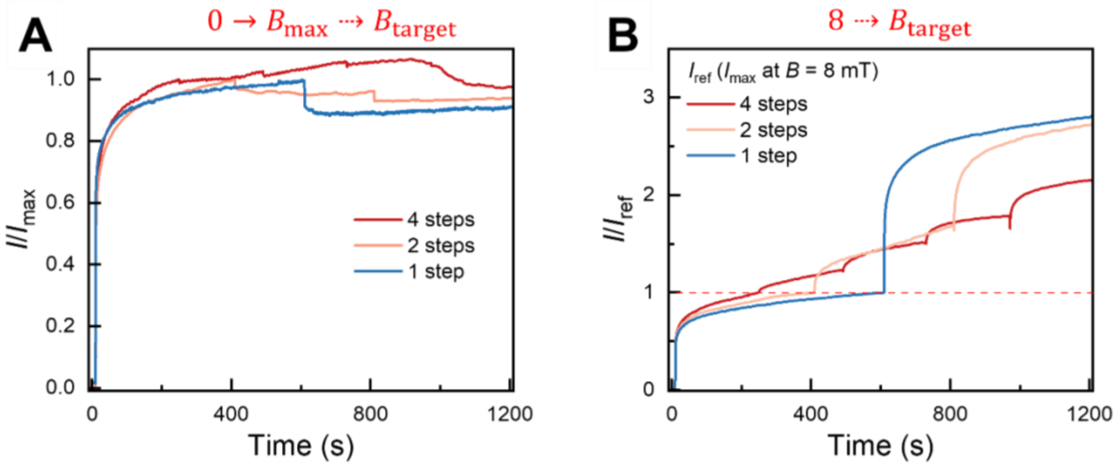

Figure S12. Suppressed current based on structural memory.

Electric characterization during the processes of (**A**) $0 \rightarrow B_{max} \dashrightarrow B_{target}$ and (**B**) $8 \dashrightarrow B_{target}$.

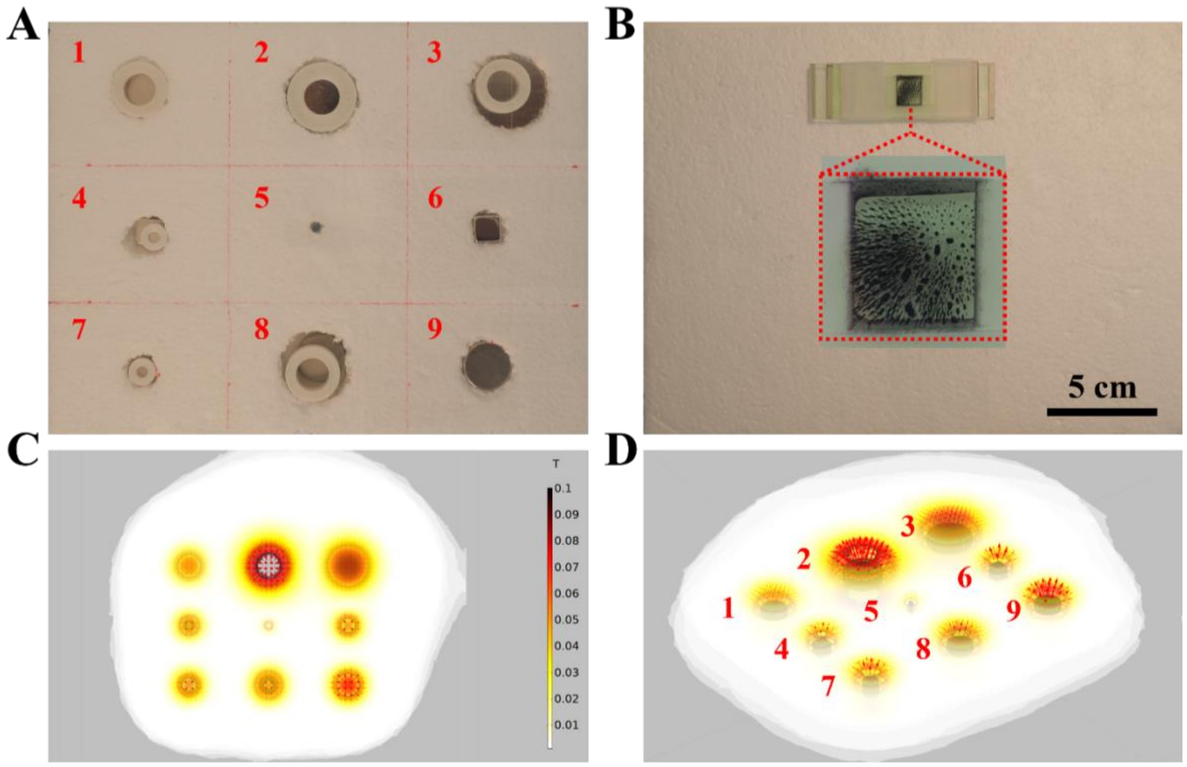

Figure S13. An artificial magnetic landscape.

A, Optical photographs of a man-made magnetic landscape comprising nine rubidium magnets in a 3×3 matrix, which are embedded in a foam board. **B**, The magnetic sensory receptor placed on the surface of the foam board. **C-D**, The simulated magnetic flux density landscape with COMSOL.

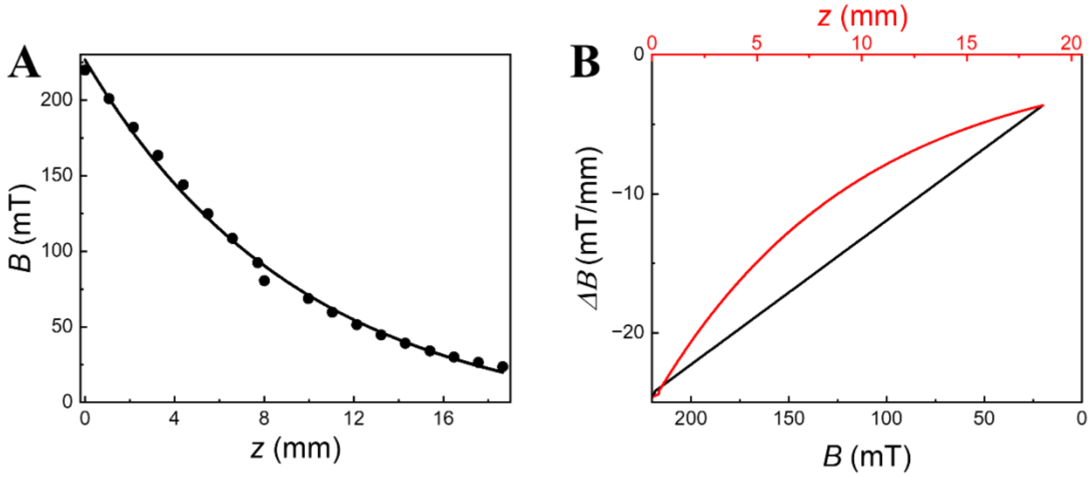

Figure S14. Magnetic fields of the typical disc magnet (magnet No. 8 and 9) used in experiments.

A, Magnetic field strength on the magnet axis as a function of distance from the surface of the magnet, showing experimentally measured data (dots) and theoretical fits (lines). **B**, The corresponding vertical field gradients $\Delta B = dB/dz$.

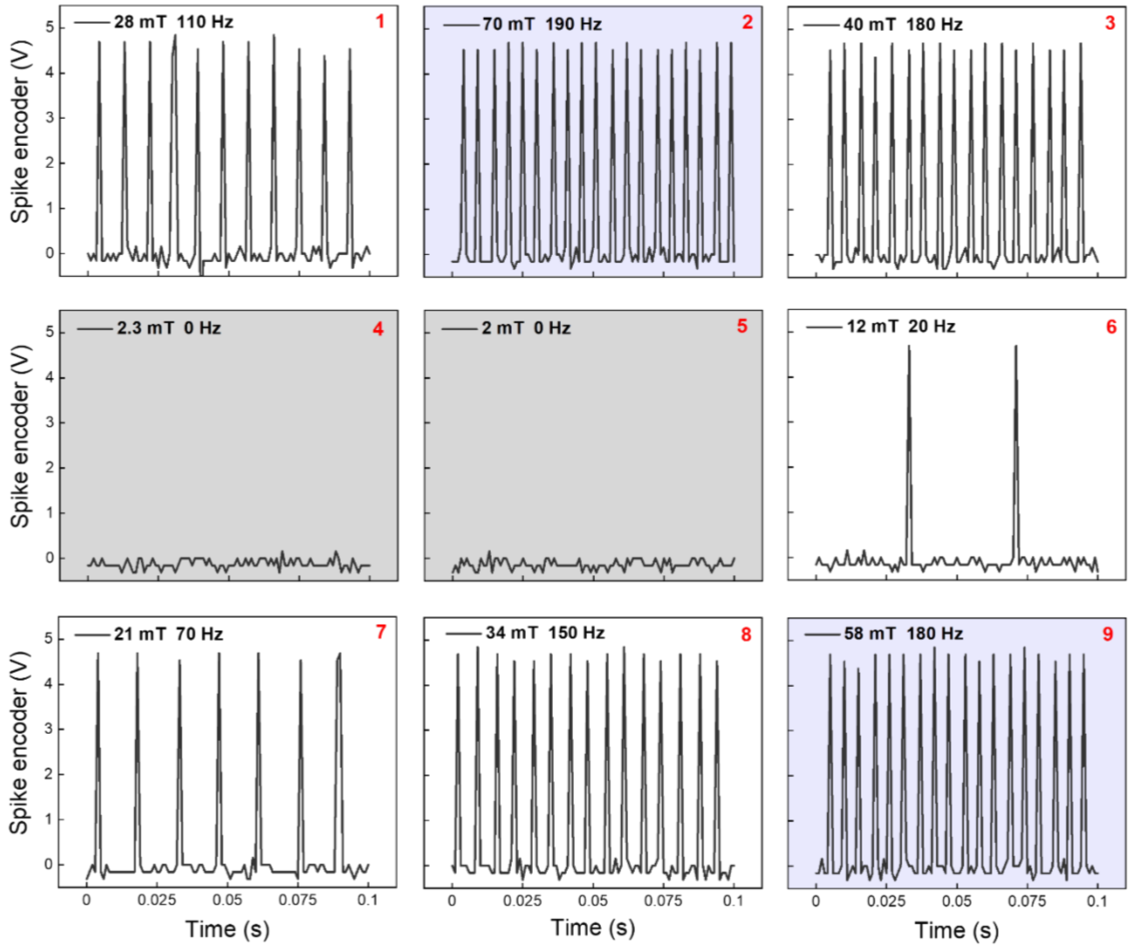

Figure S15. Mapping the magnetic landscape with the magnetic sensory system.

The fields at positions 4 and 5, and 2 and 9 cannot be perceived and discerned because they are beyond the lower and upper limits of the sensation, respectively.

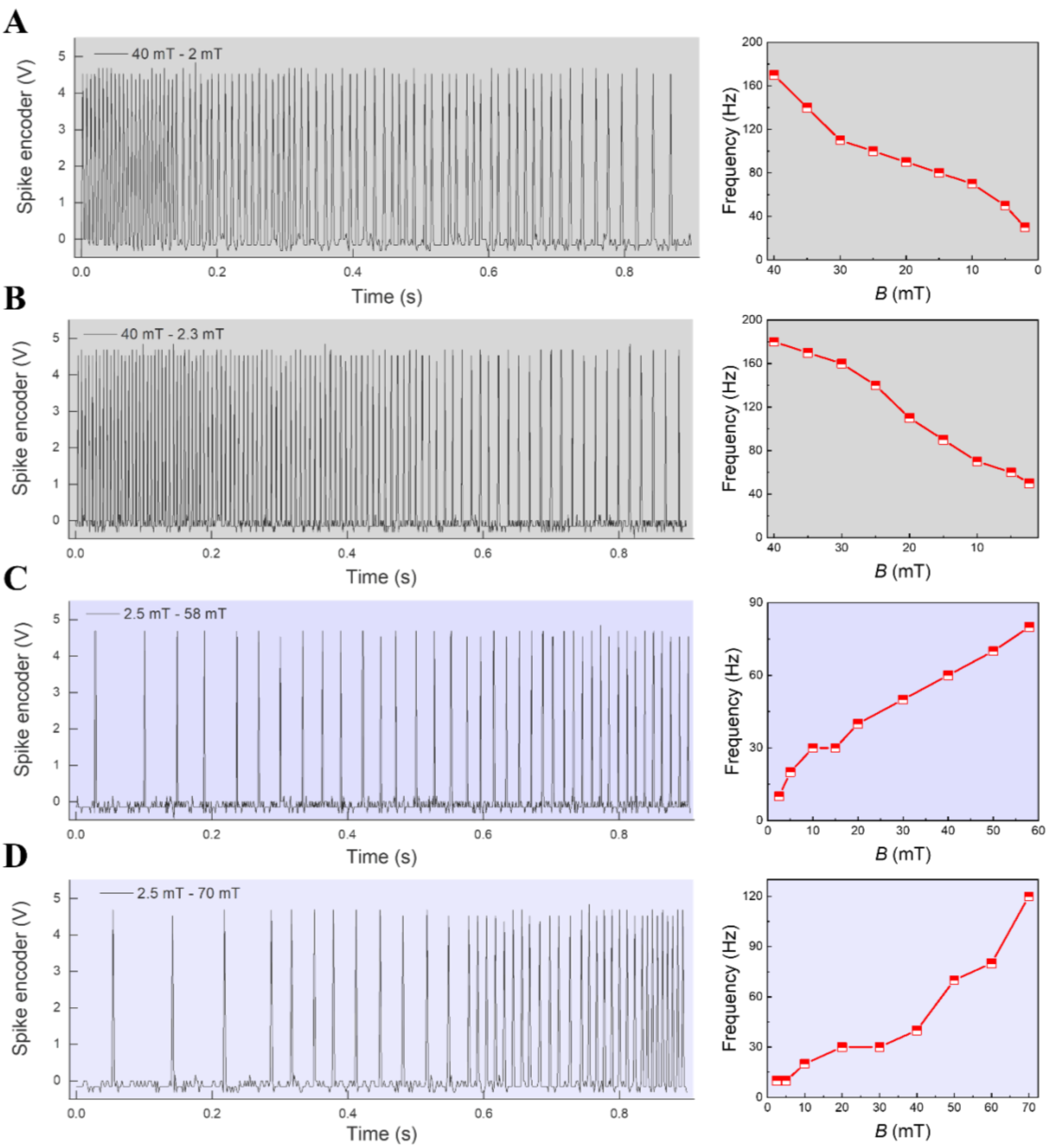

Figure S16. Magnetic sense training by promoted and depressed adaptation.

A-B, Promoting the sensory system by first approaching it to a high-strength magnetic field at 40 mT (dB/dz = 5.7 mT/mm) and then gradually to the aimed field at 2 (dB/dz = 1.7 mT/mm) (**A**) and 2.3 (dB/dz = 1.8 mT/mm) (**B**) mT, respectively. **C-D**, Depressing the sensory system by gradually approaching it to the aimed field from 2.5 (dB/dz = 1.8 mT/mm) to 58 (dB/dz = 7.6 mT/mm) (**C**) and 70 (dB/dz = 8.8 mT/mm) (**D**) mT, respectively.

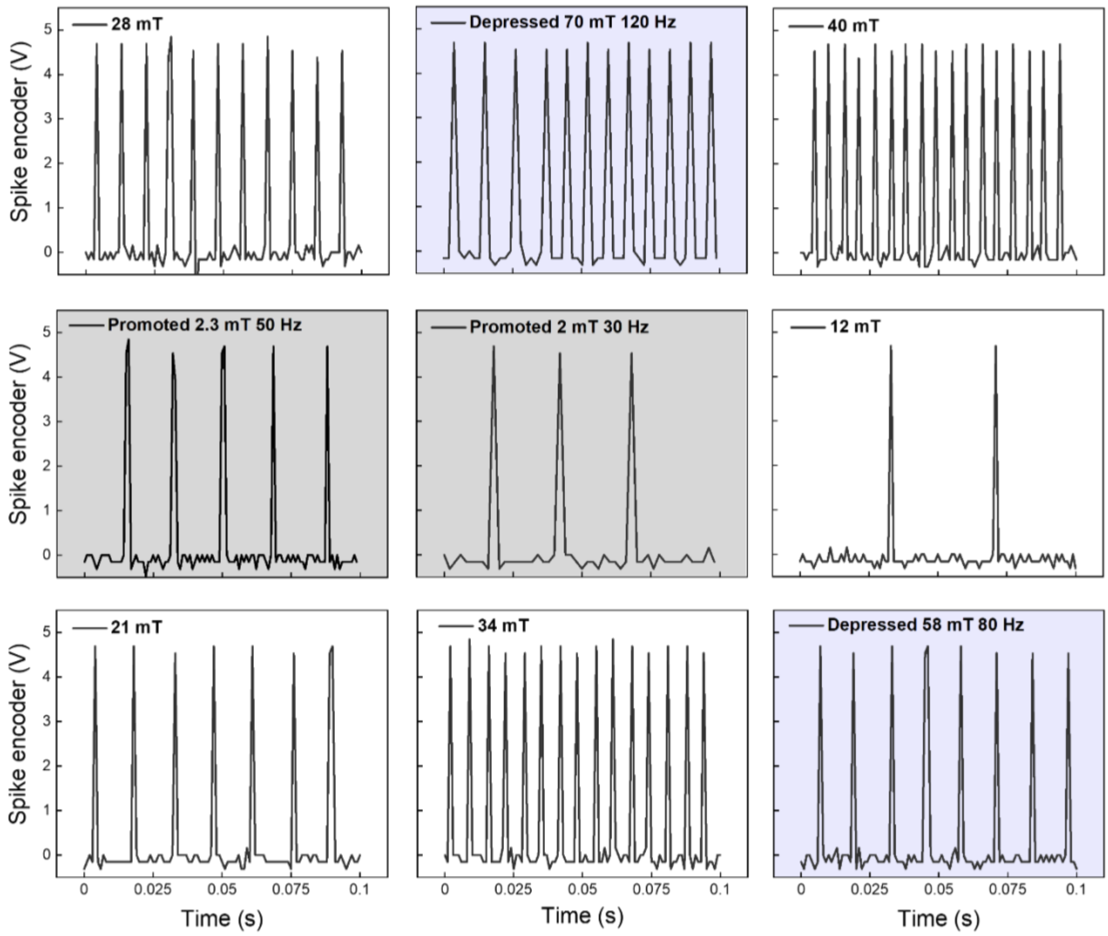

Figure S17. The restored mapping of the magnetic landscape after training.
